# Sustainable sizing, dispatch, and resilience planning of hybrid microgrids using Arctic Puffin Optimization

**DOI:** 10.1038/s41598-026-37727-0

**Published:** 2026-02-23

**Authors:** Ahmed H. Yakout, Amr S. Mashaal, Adel M. Alfons, Abdelrahman M. Metwaly, Hany M. Hasanien, Waheed Sabry, Marwa Ahmed

**Affiliations:** 1 Department of Electrical Power and Machines, Faculty of Engineering, Ain Shams University, Cairo, Egypt; 2 Department of Electrical Engineering, College of Engineering, University of Sharjah, Sharjah, 27272 United Arab Emirates; 3 Department of Electrical Power and Energy, Military Technical College (MTC), Cairo, Egypt; 4 Department of Electrical and Control, Arab Academy for Science, Technology and Maritime Transport, Cairo, Egypt

**Keywords:** Arctic Puffin Optimization (APO), Hybrid Microgrid, Multi-objective Optimization, Sizing and Dispatch, Sustainability, BESS Degradation, Resilience Analysis, Energy science and technology, Engineering

## Abstract

Hybrid microgrids combining photovoltaic (PV), wind turbine (WT), diesel generator (DG), and battery energy storage systems (BESS) provide a practical pathway for delivering reliable and low-carbon energy to isolated regions. However, their optimal sizing and dispatch planning constitute a challenging multi-objective problem due to renewable intermittency, battery degradation, and competing economic–environmental trade-offs. This paper proposes a novel Arctic Puffin Optimization (APO)-based framework for the techno-economic planning of standalone hybrid microgrids. The model simultaneously minimizes the Annual System Cost (ASC), carbon dioxide (CO_2_) emissions, and Loss of Power Supply Probability (LPSP) through integrated component sizing, dispatch optimization, and adaptive constraint handling. Two real-world case studies from Ras Ghareb, Egypt, using hourly solar, wind, and load profiles validate the proposed approach. Comparative results demonstrate that APO consistently outperforms Grey Wolf Optimizer (GWO), Ant Lion Optimizer (ALO), and Starfish Optimization Algorithm (SFOA), achieving up to 8% lower ASC, 17% higher renewable penetration, and zero LPSP while maintaining stable convergence behavior. Sensitivity analyses across varying load demands, wind speeds, irradiance levels, and generator constraints confirm the robustness of the optimized configurations. By directly incorporating emission costs and battery degradation into the objective function, the framework ensures realistic, economically viable, and environmentally responsible system design suitable for off-grid hybrid energy applications.

## Introduction

Standalone hybrid microgrids that integ rate renewable sources like solar and wind with diesel-based backup generators and energy storage units (ESS) provide a practical and eco-friendly approach for delivering power to off-grid regions. This setup helps decrease dependence on fossil fuels and mitigates greenhouse gas emissions. However, optimal sizing of their components is critical. Oversizing increases capital costs, while undersizing compromises reliability and increases reliance on fossil fuel backup. As a result, determining the most effective mix of solar panels, wind energy systems, diesel-based power units, and battery storage remains a complex task involving multiple, often conflicting, optimization objectives. Numerous metaheuristic and hybrid methods have been applied to tackle this challenge. Yet, existing approaches often struggle with high-dimensional search spaces, convergence speed, algorithmic complexity, or neglect some key performance metrics such as emissions or battery aging. This underscores the need for novel, efficient, and reliable techniques to guide real-world implementation. In recent years, hybrid microgrid systems have drawn substantial interest due to their potential to supply clean and reliable energy in remote areas. Optimization of these systems is essential, and metaheuristic algorithms have emerged as powerful tools for sizing their components. Wang et al. introduced Arctic Puffin Optimization (APO) as a promising metaheuristic optimizer for complex engineering tasks, yet it has not been validated in the context of hybrid microgrid systems, leaving its domain-specific effectiveness unverified ^1^. Singh and Kumar applied APO for optimizing hotel-scale hybrid microgrids with PV, WT, and BESS, minimizing LCOE; however, they neglected critical environmental and reliability metrics, limiting the practical scope of their analysis ^2^. Mojtaba Hadi et al. utilized a Mixed-Integer Nonlinear Programming formulation to design isolated DC microgrids for island communities, incorporating cost and autonomy objectives, but faced high computational complexity and lacked algorithmic adaptability ^3^. Zhu et al. proposed a SMOSO-based multi-objective sizing framework incorporating BESS degradation and CO$_2$ emissions using the SAMOGA algorithm, but failed to integrate diesel generator dispatch, weakening its application in off-grid resilience ^4^.Other recent works have approached the problem from different angles. Rahman et al. investigated a PV-WT-biogas-battery microgrid system with a focus on metaheuristic sizing but omitted diesel generators, which are often crucial in isolated scenarios ^5^. Conte et al. presented a hybrid microgrid optimization model with detailed energy dispatch control, yet their strategy did not utilize APO or evaluate algorithmic efficiency across multiple scenarios ^6^. Chehade and Karaki developed a BOOST decision-support system for PV-BESS design using ordinal optimization but excluded wind and diesel technologies, limiting generalizability to full hybrid systems ^7^. Another study on hybrid PV-WT-DG-BESS systems in Saudi Arabia employed the IMOEAD algorithm for sizing under cost and LPSP objectives, but lacked integration with degradation modeling ^8^.

Expanding further, Traoré et al. used enhanced genetic algorithms to size PV-WT microgrids without including dispatch strategies or diesel backup, resulting in limited system realism ^9^. An extensive comparison by Yu et al. employed Harris Hawk Optimization (HHO) for PV-WT-DG-BESS systems and benchmarked results across various algorithms, but environmental aspects were insufficiently explored ^10^. Bade et al. proposed a PSO-based multi-criteria framework integrating biomass, electrolyzers, batteries, and fuel cells, yet their model remained theoretical with minimal sensitivity analysis ^11^. Aguni Island case studies demonstrated MILP-based designs that included diesel generators and carbon emissions, though they lacked scalability across broader regions ^12^. Continuing the discussion on hybrid strategies, Ahn et al. focused on Ethiopian microgrids, balancing reliability and economic cost, yet failed to incorporate emissions data, weakening sustainability assessments ^13^. Yin et al. optimized hybrid systems for remote communities, but their work did not include degradation costs, making lifecycle analysis incomplete ^14^. Grasshopper Optimization Algorithm was applied to rural microgrid sizing including biomass, but lacked dispatch modeling for variable conditions ^15^. Another study on fuel cell-integrated microgrids by Bade et al. introduced novel configurations but did not benchmark against standard algorithms like APO or GWO ^16^. In addition to these algorithmic strategies, newer algorithmic variants have emerged. Advanced algorithmic developments such as APO-JADE hybrids showed improved search balance in test cases but lacked deployment in real energy systems ^17^. The ETAAPO variant introduced tangent flight and mutation operators to enhance global search, but has not yet been tested on hybrid microgrid configurations ^18^. ACS Omega highlighted the application of APO in voltage control, but these efforts were limited to DC systems and did not address energy resource planning ^19^. Energy dispatch optimization is a related yet distinct area of research. Li et al. employed a chance-constrained MILP scheduling method under uncertainty but assumed fixed system sizes, thereby decoupling dispatch from sizing ^20^. In a follow-up, Li et al. proposed a multi-objective dispatch approach prioritizing user comfort, still failing to include infrastructure sizing in the loop ^21^. Other models focused on dispatch-only optimization without re-evaluating component sizing under variable operational profiles ^22^. Some studies have attempted to bridge the gap between sizing and dispatch. A Scientific Reports study proposed a demand response (DR) linked microgrid, but its applicability was confined to grid-connected setups ^23^. Nature published a Dandelion Algorithm-based approach for demand-side management; however, the system lacked standalone renewable energy modeling ^24^. GWO and Cuckoo Search hybrids showed promise in component sizing but omitted emissions, weakening environmental impact analysis ^25^. Carbon-focused optimization for university campuses incorporated CO$_2$ metrics in cost functions but lacked comparative algorithmic benchmarking ^26^. MDPI Electronics outlined a multi-objective AC/DC microgrid sizing scheme without hybrid dispatch logic, resulting in suboptimal system performance ^27^. Wiley’s grid-connected HRES model emphasized reliability but did not consider off-grid deployment constraints ^28^. Review and synthesis studies help highlight existing gaps. A ScienceDirect review pointed out the gap in algorithm scalability under real-world constraints ^29^. E3S conference proceedings criticized LPSP and cost-based algorithms for failing to balance objectives efficiently ^30^. ResearchGate reviews noted the widespread absence of emission-reliability trade-offs in unified optimization models ^31^. Another synthesis emphasized the lack of integration between sizing and energy management under uncertainty ^32^. Degradation and emission modeling remain underexplored. POST-OPT BESS degradation frameworks considered lifecycle losses but were restricted to PV-BESS systems only, ignoring DG and WT behavior ^33^. SMOSO included degradation and emissions in objective functions but omitted dispatch realism ^34^. A resilience-based dispatch model emphasized fault-tolerance but operated with fixed capacities, missing co-optimization potential ^35^. A campus-level hybrid optimization study repeated sizing assumptions across conditions, lacking adaptive control mechanisms ^36^. Literature on voltage regulation and resilience stressed the need for integrated sizing and dispatch approaches, yet no concrete frameworks were presented ^37^. To broaden this foundation, other studies have introduced hybrid methods that bring diverse perspectives. Hossain et al. developed a fuzzy-logic integrated PSO algorithm for optimizing PV-WT-DG hybrid systems under stochastic conditions, but it did not factor in environmental emissions or long-term degradation ^38^. Alami et al. used the Whale Optimization Algorithm (WOA) for microgrid sizing in North African communities; however, their model neglected resilience metrics and failed under high demand volatility ^39^. Lastly, a study by Afolabi et al. employed Firefly Optimization for rural electrification but lacked proper validation against real-world energy use profiles and omitted diesel-based backups ^40^. In related algorithmic developments, Paul et al. ^41^ applied a quasi-oppositional-based Artificial Rabbit Optimization (ARO) method for optimal PMU placement in wide-area monitoring systems of transmission networks. Their work demonstrates how recent metaheuristic innovations can effectively address complex, high-dimensional power system problems. While such studies highlight the growing relevance of nature-inspired optimizers in power system analysis, they primarily focus on transmission-level observability rather than integrated energy resource planning.

Complementing these efforts, Hazra et al. ^42^ explored moth–flame optimization (MFO) for coordinating large-scale tidal–solar–wind–hydro–thermal systems with electric vehicle participation. Their results show that MFO can efficiently manage multi-source coordination and reduce operational emissions through optimized dispatch and EV charging strategies. Although these studies confirm the strength of advanced metaheuristics such as ARO and MFO in diverse power system applications, they mainly address short-term operational scheduling and monitoring tasks. In contrast, the present work advances long-term techno-economic sizing and dispatch co-optimization of standalone PV–WT–DG–BESS microgrids, where Arctic Puffin Optimization (APO) provides notable improvements in convergence stability, solution robustness, and integration of cost, emissions, degradation, and reliability considerations.

In addition to traditional metaheuristics, a series of recent studies has demonstrated a growing interest in Arctic Puffin Optimization (APO) for advanced power and energy applications. Lv et al. ^43^ applied APO for parameter identification of doubly fed induction generator controllers, while Abaza et al. ^44^ used APO for optimal transformer circuit parameter extraction with experimental verification. Enhanced APO variants have also been investigated for electrochemical systems, such as PEM fuel cell parameter estimation ^45^. Beyond energy systems, APO has shown strong adaptability in complex multi-agent and nonlinear environments, including 3D UAV swarm trajectory planning ^46^ and production models under uncertainty incorporating sustainability metrics ^47^. These recent developments confirm APO’s growing relevance and demonstrate its robustness across diverse engineering domains. However, despite these advances, APO has not yet been employed for long-term techno-economic sizing and dispatch co-optimization of standalone PV–WT–DG–BESS microgrids, highlighting a key research gap addressed by the present study.

To synthesize the insights from prior work, Tables 1 and 2 summarize the optimization methods, application areas, and methodological trade-offs reported in recent microgrid studies. Despite this progress, substantial gaps remain–particularly the lack of any framework employing Arctic Puffin Optimization (APO) for the full techno-economic co-optimization of PV–WT–DG–BESS systems under real meteorological and demand conditions. Existing APO-related studies focus mainly on control problems or single-objective formulations and do not integrate capacity sizing with dispatch decisions, nor do they jointly consider cost, emissions, battery degradation, and reliability in a unified model. Comparative benchmarking of APO against established methods such as GWO, ALO, and SFOA is also scarce, and most prior works treat sizing and operation separately, limiting their ability to capture realistic interactions between renewable availability and load variability. Motivated by these gaps, this study develops a unified APO-based framework that simultaneously determines optimal system sizing and dispatch while minimizing annualized cost, CO_2_ emissions, and Loss of Power Supply Probability (LPSP), with degradation and environmental penalties embedded directly in the objective function. The proposed method is validated using real seasonal data from Ras Ghareb, Egypt, supported by extensive sensitivity analysis under varying load and renewable conditions, and benchmarked against GWO, ALO, and SFOA to demonstrate APO’s robustness, convergence performance, and comparative advantages.Finally, the structure of this paper is as follows: Section 1 introduces the research motivation and literature gaps; Section 2 presents the system modeling; Section 3 describes the control and dispatch strategy; Section 4 formulates the optimization problem; Section 5 details the APO algorithm; Section 6 reports the simulation results, comparative evaluation, and sensitivity analysis; Section 7 discusses the study’s limitations and scope for future work; and Section 8 provides the concluding remarks.

**Table 1:** Summary of selected literature and addressed limitations.

Refs.	Authors/Source	Method	Focus area	Key advantage	Limitation addressed by this work
^2^	Kassab et al	Metaheuristics	Sizing	Cost-reliability trade-off	Unstable under dynamic load conditions
^4^	Madathil et al	Rolling Horizon	Sizing + Resilience	Resilience-aware microgrid design	Too complex for real-time deployment
^5^	Wüstenhagen et al	PSO	Sizing	Simple and fast search	Trapped in local minima
^6^	Wang et al	APO	Algorithm	Novel optimizer with good exploration	Not applied to hybrid MG systems
^7^	APO–JADE (MDPI)	APO + JADE	Optimization	Improved convergence	No real-world validation in energy domain
^9^	SMOSO	SAMOGA	Degradation + Emission	Incorporates battery aging and C_2_ penalty	Ignores diesel generator operation
^10^	Li et al	MILP	Control	Uncertainty-aware dispatch	Sizing and control not linked
^12^	Nature (2024)	Dandelion Algorithm	DSM (Grid-Connected)	Effective demand-side management	No modeling of standalone renewables
^13^	POST-OPT BESS	Heuristic	Degradation Modeling	Lifecycle-aware battery sizing	Focused only on PV–BESS
^14^	Voltage Resilience Review	Thematic Review	Resilience + Control	Identifies key integration needs	No implementation or validation
^16^	Uncertainty Sizing Study	Metaheuristics	Uncertainty Modeling	Incorporates renewable resource variability	Misses real-time dispatch linkage
^17^	Optimal HRES (2023)	Joint PV-WT-BESS-DG	Sizing + Economics	Full hybrid model	Weak dispatch/control layer
^21^	APO DVR Control	APO	Voltage Regulation	Novel APO use in control systems	Does not address sizing
^25^	Hybrid GWO-Cuckoo	Metaheuristic Hybrid	Sizing	Combines GWO and CS for stronger performance	Lacks emissions evaluation
^40^	Resilience Review (2023)	Review	Sizing + Dispatch	Calls for integrated optimization	No demonstration or case study

**Table 2:** Comparison of optimization techniques used in microgrid design.

Technique	Strengths	Weaknesses
PSO	Simple to implement, fast convergence, widely used in hybrid microgrid systems	Prone to local optima, less effective in handling multi-objective, high-dimensional problems
GA	Flexible genetic operators, adaptable to various system configurations	Slower convergence, sensitive to parameter settings
GWO	Balanced exploration–exploitation, effective for nonlinear objectives	Requires fine-tuning, performance drops with increased complexity
ALO	Good global search inspired by swarm behavior	Weaker under uncertainty and noise, slower convergence
APO	Strong global search, avoids premature convergence	Limited testing in microgrids, lacks dispatch–sizing integration
Hybrid (e.g., GWO+CS, APO–JADE)	Merges strengths of base algorithms, improves convergence and diversity	Complex tuning, higher computation overhead
MILP	Deterministic and scalable for linear control models	Inflexible for nonlinear or uncertain systems
Stochastic (e.g., SMOSO)	Captures uncertainty, aging, and CO$_2$ penalties	High computational cost, rarely tested in real cases

## Microgrid components

**Fig. 1:**
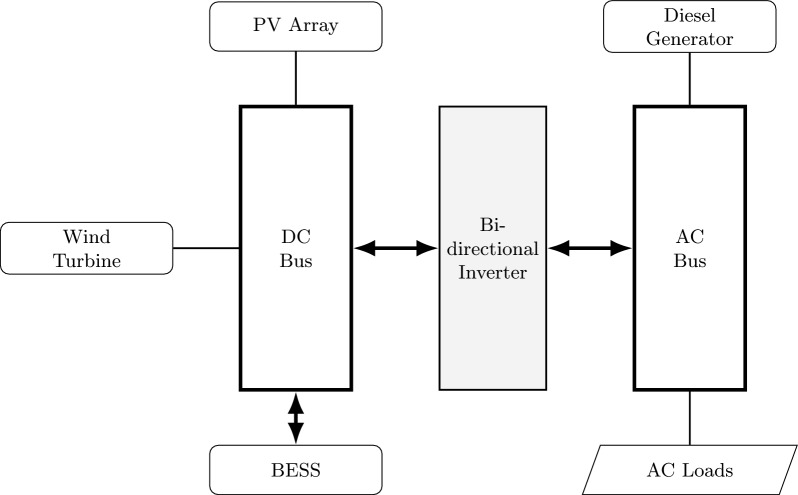
Block diagram of the proposed hybrid AC/DC microgrid architecture.

An isolated hybrid microgrid relies on the synergistic operation of multiple energy sources and storage units to provide a reliable and sustainable power supply under varying environmental conditions. This work models a hybrid energy system comprising four key elements: solar PV modules, wind energy converters, a backup diesel unit, and a battery-based energy storage solution. The system architecture includes both AC and DC buses–where the diesel unit and electrical loads are tied to the AC bus, while the renewable sources and storage components interface with the DC bus.A bidirectional inverter facilitates power exchange between these buses, enabling flexible energy management. This section details the physical principles, mathematical models, and operational roles of each component within the integrated microgrid.Table 3 presents the technical and economic parameters used for each microgrid component. The capital costs reflect typical market prices, with the diesel generator exhibiting significantly higher upfront investment due to its capacity and complexity. Rated powers for PV and wind systems align with small- to medium-scale installations, while operational lifetimes vary between 10 to 25 years, consistent with industry standards. The wind turbine cut-in and cut-out speeds indicate its operational wind speed range, and battery state-of-charge limits ensure safe and efficient storage management. These values were selected to realistically model the microgrid’s performance and economic feasibility. Figure 1 presents the structural layout of the proposed hybrid microgrid, including generation sources, storage, and load connections.

### Photovoltaic (PV) system

Photovoltaic panels convert incident solar irradiance into electrical energy via semiconductor effects, with output power directly influenced by solar radiation intensity and cell temperature. Given the intermittent nature of solar energy, accurately modeling the PV output is critical for predicting system performance and optimizing component sizing.

The instantaneous electrical power generated by the PV array at time $t$ is modeled as:

1 $$\begin{aligned} P_{\textrm{PV}}(t) = N_{\textrm{PV}}\, \eta _{\textrm{PV}}\, P_{\textrm{PV}}^{\textrm{rated}} \left( \frac{I(t)}{I_{\textrm{ref}}} \right) \left[ 1 - \gamma \big ( T_{\textrm{cell}}(t) - T_{\textrm{ref}} \big ) \right] \end{aligned}$$

The cell temperature is estimated empirically as:

2 $$\begin{aligned} T_{\textrm{cell}}(t) = T_{\textrm{ambient}}(t) + 0.0254\, I(t) \end{aligned}$$

In these expressions, $N_{\textrm{PV}}$ is the number of installed PV modules, $\eta _{\textrm{PV}}$ is the module efficiency under standard test conditions (STC), and $P_{\textrm{PV}}^{\textrm{rated}}$ is the rated module power at STC. The term *I*(*t*) denotes the solar irradiance at time *t* (W/m$^{2}$), while $I_{\textrm{ref}} = 1000$ W/m$^{2}$ is the reference irradiance. The temperature coefficient $\gamma$ quantifies PV power reduction per degree Celsius above the reference temperature $T_{\textrm{ref}} = 25^{\circ }\textrm{C}$. The PV cell temperature $T_{\textrm{cell}}(t)$ is estimated from the ambient temperature $T_{\textrm{ambient}}(t)$ and irradiance using the empirical linear relation in (2).

### Wind turbine system

Wind turbines convert kinetic energy of the wind into electrical power within defined operational wind speed limits. Modeling turbine output as a function of wind speed is crucial due to wind’s nonlinear and intermittent behavior.

The wind power output at time *t* is modeled as:

3 $$\begin{aligned} P_{\textrm{wind}}(t) = {\left\{ \begin{array}{ll} 0, & v(t)< v_{\mathrm {cut\text{- }in}} \ \text {or}\ v(t) > v_{\mathrm {cut\text{- }out}}, \\[4pt] P_{\textrm{partial}}(t), & v_{\mathrm {cut\text{- }in}} \le v(t) < v_{\textrm{rated}}, \\[4pt] N_{\textrm{WT}}\,\eta _{\textrm{WT}}\,P_{\textrm{WT}}^{\textrm{rated}}, & v_{\textrm{rated}} \le v(t) \le v_{\mathrm {cut\text{- }out}}. \end{array}\right. } \end{aligned}$$

The turbine operates in partial-load mode according to:

4 $$\begin{aligned} P_{\textrm{partial}}(t) = N_{\textrm{WT}}\,\eta _{\textrm{WT}}\,P_{\textrm{WT}}^{\textrm{rated}} \frac{\,v(t)^2 - v_{\mathrm {cut\text{- }in}}^2\,}{\,v_{\textrm{rated}}^2 - v_{\mathrm {cut\text{- }in}}^2\,}. \end{aligned}$$

Here, *v*(*t*) is the wind speed at time *t*, while $v_{\mathrm {cut\text{- }in}}$, $v_{\textrm{rated}}$, and $v_{\mathrm {cut\text{- }out}}$ denote the cut-in, rated, and cut-out wind speeds, respectively. $N_{\textrm{WT}}$ is the number of turbines, $\eta _{\textrm{WT}}$ is the turbine efficiency, and $P_{\textrm{WT}}^{\textrm{rated}}$ is the rated power of a single wind turbine.

### Diesel generator

The diesel generator serves as a dispatchable power source, especially during renewable energy shortfalls or peak demand periods. Its fuel consumption is approximated by:

5 $$\begin{aligned} C(t) = a\, P_{\textrm{DG}}(t) + b\, P_{\textrm{DG}}^{\textrm{rated}} \end{aligned}$$

where *C*(*t*) denotes the instantaneous fuel consumption, $P_{\textrm{DG}}(t)$ is the generator output power at time *t*, and $P_{\textrm{DG}}^{\textrm{rated}}$ is the rated generator capacity. The coefficients $a = 0.246$ and $b = 0.08415$ (L/kWh) are empirically derived fuel parameters commonly used in microgrid diesel modeling.

### Battery energy storage system (BESS)

The BESS mitigates the temporal mismatch between generation and load by storing excess energy during surplus periods and supplying power during shortages. The battery state of charge (SOC) evolves according to the operating mode.

The SOC at time *t* is defined as:

6 $$\begin{aligned} \textrm{SOC}(t) = \frac{C(t)}{C_{\textrm{batt}}} \end{aligned}$$

During charging, the SOC increases as:

7 $$\begin{aligned} \textrm{SOC}(t) = \textrm{SOC}(t-1)(1 - \sigma ) + \frac{\eta _{\textrm{batt}}}{\eta _{\textrm{inv}}}\, E_{\textrm{charge}}(t) \end{aligned}$$

During discharging, the SOC decreases as:

8 $$\begin{aligned} \textrm{SOC}(t) = \textrm{SOC}(t-1)(1 - \sigma ) - \frac{E_{\textrm{discharge}}(t)}{\eta _{\textrm{batt}}\, \eta _{\textrm{inv}}} \end{aligned}$$

Here, *C*(*t*) is the stored battery energy at time *t*, $C_{\textrm{batt}}$ is the nominal battery capacity, $\sigma$ is the hourly self-discharge rate, $\eta _{\textrm{batt}}$ is the battery round-trip efficiency, $\eta _{\textrm{inv}}$ is the inverter efficiency, and $E_{\textrm{charge}}(t)$ and $E_{\textrm{discharge}}(t)$ denote the charging and discharging energies over the time step, respectively.

### System integration

The overall power balance ensuring load demand satisfaction at time $t$ is:

9 $$\begin{aligned} {\begin{matrix} P_{\textrm{load}}(t) =\ & P_{\textrm{PV}}(t) + P_{\textrm{wind}}(t) \\ & + P_{\textrm{DG}}(t) + P_{\textrm{BESS}}(t) \end{matrix}} \end{aligned}$$

where $P_{\textrm{BESS}}(t)$ is positive during discharge and negative during charging. The presented models balance physical accuracy and computational tractability, enabling their seamless integration within metaheuristic optimization frameworks such as the Arctic Puffin Optimization algorithm. While these deterministic models capture the core dynamics necessary for system design and control, future work can incorporate environmental variability, component aging, and stochastic disturbances to enhance realism. This modeling foundation supports reliable sizing, operational planning, and decision-making for isolated microgrids under diverse climatic and load conditions.

**Table 3:** Component Parameters and Values.

Component	Parameter	Value
PV	Capital Cost	200 USD
	Rated Power	260 W
	Lifetime	25 years
Wind	Capital Cost	700 USD
	Rated Power	1500 W
	Rated Speed	12 m/s
	Lifetime	25 years
	Cut-in Speed	0.5 m/s
	Cut-out Speed	13.5 m/s
DG	Capital Cost	14,820 USD
	Fuel Cost	1.2 USD/L
	DG Reliability	90%
	Lifetime	20 years
Battery	Capital Cost	200 USD
	Lifetime	10 years
	Min. SOC	20%
	Max. SOC	90%
Project	Lifetime	20 years
	Interest Rate	7%

## Control strategy

To ensure a continuous and reliable power supply in an isolated microgrid subject to variable renewable generation, an adaptive control strategy is developed. This strategy dynamically manages the power flows among photovoltaic (PV) arrays, wind turbines, a battery energy storage system (BESS), and a diesel generator (DG). Control decisions are based on the instantaneous power balance and the battery’s state of charge (SOC), with the objective of minimizing fuel consumption and maximizing the use of renewable energy.

At each time step *t*, the net power balance is computed as:

10 $$\begin{aligned} \Delta P(t) = P_{\text {RE}}(t) - P_{\text {load}}(t) \end{aligned}$$

where $P_{\text {RE}}(t) = P_{\text {solar}}(t) + P_{\text {wind}}(t)$ represents the total renewable generation, and $P_{\text {load}}(t)$ is the electrical load demand.

### Case I: renewable generation $\ge$ load demand

When $\Delta P(t) \ge 0$, renewable generation is sufficient to meet or exceed the load. The system responds as follows:

- If $\Delta P(t) = 0$, the generation exactly matches the load, and the BESS remains idle.
- If $\Delta P(t) > 0$ and $SOC(t) < SOC_{\max }$, the surplus power is directed to charge the battery.
- If $\Delta P(t) > 0$ and $SOC(t) = SOC_{\max }$, the battery is fully charged, and the excess energy is curtailed.

### Case II: renewable generation < load demand

When $\Delta P(t) < 0$, a power shortfall occurs. The control system initially attempts to compensate by discharging the battery, with the required energy given by:

11 $$\begin{aligned} E_{\text {batt}}(t) = |\Delta P(t)| \cdot \Delta t \end{aligned}$$

If the battery can supply this energy, the DG remains inactive. Otherwise, the DG is dispatched under two possible conditions:

#### Case II-a: deficit below minimum DG output

If the energy shortfall is smaller than the DG’s minimum power threshold $P_{\text {DG,min}}$, the DG operates at $P_{\text {DG,min}}$, and the excess energy is stored:

12 $$\begin{aligned} E_{\text {DG}}(t) = |\Delta P(t)| \cdot \Delta t + E_{\text {batt}}(t) \end{aligned}$$

#### Case II-b: deficit exceeds minimum DG output

The system then evaluates whether the battery alone can supply the load:

13 $$\begin{aligned} E_{\text {batt}}(t) \ge |\Delta P(t)| \cdot \Delta t \end{aligned}$$

If so, the battery covers the full deficit. Otherwise, the battery and DG share the load:

14 $$\begin{aligned} |\Delta P(t)| \cdot \Delta t = E_{\text {batt}}(t) + E_{\text {DG}}(t) \end{aligned}$$

**Battery SOC Dynamics** The battery’s state of charge (SOC) is updated dynamically to reflect energy storage or withdrawal. These updates account for conversion efficiencies and battery degradation. In the control framework, $\Delta P(t)$ represents the net power balance at time *t*, calculated as the difference between total renewable generation and the load demand. The total renewable energy $P_{\text {RE}}(t)$ is the sum of solar generation $P_{\text {solar}}(t)$ and wind generation $P_{\text {wind}}(t)$, while $P_{\text {load}}(t)$ denotes the total electricity required by consumers at time *t*. The diesel generator is constrained by a minimum operating threshold $P_{\text {DG,min}}$, below which it cannot function efficiently. When renewable sources are insufficient, the battery system contributes an amount of energy $E_{\text {batt}}(t)$ to cover the deficit, and if needed, the diesel generator supplies energy denoted by $E_{\text {DG}}(t)$. During surplus conditions, the battery can absorb energy $E_{\text {charge}}(t)$, while $E_{\text {discharge}}(t)$ refers to the energy withdrawn from the battery during power shortages. The battery’s performance is influenced by its round-trip efficiency $\eta _{\text {batt}}$, and the inverter efficiency $\eta _{\text {inv}}$, both of which account for energy losses during conversion. The battery also experiences a natural self-discharge over time, modeled by the rate $\sigma$. The total energy storage capacity of the battery bank is represented by $C_{\text {total}}$, and its dynamic state is described by the state of charge *SOC*(*t*), which evolves with charging, discharging, and self-discharge processes. Each update occurs over a discrete time interval $\Delta t$, representing the simulation or control time step.

## Objective function

The optimization strategy seeks to minimize both the Annual System Cost (ASC) & $\text {CO}_{2}$ emissions, while ensuring that the system operates reliably and efficiently. Key operational requirements–such as maintaining power availability, increasing reliance on renewable sources, and minimizing surplus energy–are embedded in the objective function using a penalty-based formulation. This mechanism discourages solutions that violate critical performance thresholds.

Annual System Cost (ASC), Excess Energy Ratio (EER), Renewable Fraction (RF), and Loss of Power Supply Probability (LPSP) are used throughout the analysis and are defined at their first appearance for clarity.

The optimization problem is expressed as a multi-objective function defined below:

15 $$\begin{aligned} \min F^*(X) = w_1 \cdot ASC(X) + w_2 \cdot CO_2(X) + P_{\text {penalty}}(X) \end{aligned}$$

where $F^*(X)$ represents the overall objective function to be minimized, combining economic and environmental goals; $w_1$ and $w_2$ are the weighting factors for the Annual System Cost and carbon emissions, respectively; and $P_{\text {penalty}}(X)$ denotes the penalty applied to infeasible solutions that violate system constraints.

The weighting factors $w_{1}=0.6$ and $w_{2}=0.4$ were selected to reflect common practice in hybrid microgrid planning, where economic viability typically receives slightly higher priority than emission reduction, particularly in remote or off-grid regions with constrained budgets. Several recent studies have adopted similar weight distributions when balancing economic and environmental objectives. Moreover, preliminary simulations conducted in this work showed that assigning a higher weight to cost leads to more stable and feasible system configurations without compromising renewable penetration. To further ensure robustness, additional tests with alternative weight combinations (e.g., 0.5/0.5 and 0.7/0.3) yielded similar optimal configurations and performance trends, confirming that the final results are not overly sensitive to the chosen weighting scheme.

The penalty term $P_{\text {penalty}}$ accounts for constraint violations and is defined as:

16 $$\begin{aligned} P_{\text {penalty}}(X) = \sum _{j=1}^{n} \lambda _j \,\max \!\left( 0,\, g_j(X)\right) , \end{aligned}$$

where $g_j(X)$ represents the violation of constraint *j* (dimensionless), and $\lambda _j$ is a dimensionless penalty coefficient that controls the severity of the penalization applied to infeasible solutions.

### Annual system cost (ASC)

The *Annual System Cost* is composed of five cost elements: capital, replacement, operation and maintenance, fuel, and emissions. It is expressed as:

17 $$\begin{aligned} ASC = ACC + ARC + AOM + AFC + AEC \end{aligned}$$

**1. Annual Capital Cost (ACC)**

The Annual Capital Cost accounts for the amortized investment cost of the system components and is calculated using the Capital Recovery Factor (CRF):

18 $$\begin{aligned} ACC = CRF(ir, y) \cdot C_{\text {capital}} \end{aligned}$$

19 $$\begin{aligned} CRF(ir, y) = \frac{ir \cdot (1 + ir)^y}{(1 + ir)^y - 1} \end{aligned}$$

where *ir* is the interest rate and *y* is the expected lifetime of the component.

**2. Annual Replacement Cost (ARC)**

The Annual Replacement Cost accounts for the future replacement of system components such as batteries, inverters, or diesel generators that have shorter lifespans than the overall project. It ensures that the financial planning includes the cost of purchasing new equipment at the end of each component’s useful life. To compute ARC, the Sink Fund Factor (SFF) is used. The SFF spreads the future replacement cost over the project lifetime by calculating the fixed annual amount needed to accumulate the replacement cost over a given number of years with interest:

20 $$\begin{aligned} ARC&= SFF(ir, y_{\text {rep}}) \cdot C_{\text {replacement}}\end{aligned}$$

21 $$\begin{aligned} SFF(ir, y_{\text {rep}})&= \frac{(1 + ir)^{y_{\text {rep}}} - 1}{ir} \end{aligned}$$

The yearly cost for operating and maintaining the system is determined by applying a predetermined fraction of the upfront cost associated with each component. This percentage reflects expected routine expenses such as inspections, preventive maintenance, minor repairs, spare parts, and labor. The total estimated O&M cost is then evenly distributed over the useful life of the component to provide a consistent annual value. These estimates are typically derived from manufacturer data, empirical field reports, or standardized cost ratios established in literature or industry guidelines. This approach ensures that recurring operational expenses are realistically incorporated into the system’s annual financial assessment, supporting long-term sustainability and reliability.

**3. Annual Fuel Cost (AFC)**

The Annual Fuel Cost (AFC) reflects the total monetary expenditure associated with the diesel fuel consumed by the generator over the course of a year. It is computed by multiplying the unit cost of diesel fuel by the cumulative fuel consumption across all time intervals. This calculation captures the generator’s role in meeting power demand during periods when renewable sources or battery storage are insufficient. The fuel consumption at each time step depends on the generator’s loading conditions and efficiency characteristics, which are often nonlinear and vary with operating power. By summing the fuel use over 8,760 hours (one full year), the AFC provides a realistic estimate of recurring fuel expenses, which is crucial for assessing both the economic and environmental impacts of diesel generator operation within the hybrid energy system.

22 $$\begin{aligned} AFC = C^* \cdot \sum _{t=1}^{8760} F^*(t) \end{aligned}$$

where $C^*$ is the unit price of diesel (per liter), and $F^*(t)$ denotes the fuel consumption at time *t*.

**4. Annual Emission Cost (AEC)**

The Annual Emission Cost (AEC) quantifies the environmental impact associated with operating the diesel generator, specifically the cost of carbon dioxide CO$_2$ emissions. This metric translates environmental externalities into monetary terms, allowing the optimization framework to penalize carbon-intensive energy generation and favor cleaner alternatives.

The AEC is calculated using the following expression:

23 $$\begin{aligned} AEC = \frac{E_f \cdot E_{fc} \cdot \sum _{t=1}^{8760} P_{DG}(t)}{1000} \end{aligned}$$

Here, $E_f$ is the emission factor (kg CO$_2$/kWh), $E_{fc}$ is the emission cost factor (cost per ton of CO$_2$), and $P_{DG}(t)$ is the generator output at time *t*.

The economic parameters used in this study are based on regionally consistent and literature-supported values. The diesel fuel price (0.95–1.20 USD/L) reflects the prevailing subsidized rate for industrial and remote-area supply reported by the Egyptian Ministry of Petroleum and Mineral Resources (2023). The emission cost (25 USD/ton of CO$_2$) falls within the typical range adopted in recent Middle Eastern and African microgrid studies (15–40 USD/ton) in regions without regulated carbon markets. The interest rate (7–10%) corresponds to Central Bank of Egypt infrastructure financing rates during 2023–2024. These references ensure that the cost and environmental assumptions used in the ASC and emission models reflect realistic and region-appropriate economic conditions.

The optimization process is subject to several technical constraints. First, the LPSP must not exceed a predefined reliability threshold to ensure consistent power delivery. Second, the Fractional Renewable (FR) must exceed a specified minimum value to promote renewable energy usage. Third, the Excess Energy Ratio (EER) must remain within acceptable bounds to prevent system overdesign and energy spillage. Any violation of these constraints invokes the penalty term described earlier, effectively steering the optimization away from infeasible configurations.

By integrating cost minimization, emission reduction, and constraint enforcement into a unified formulation, this objective function enables a robust and comprehensive design strategy for isolated hybrid microgrid systems.

### Optimization constraints

To ensure a technically sound and practically feasible microgrid design, the optimization framework incorporates a set of critical system-level constraints. These constraints reflect real-world reliability, operational limits, and energy utilization efficiency. hese criteria are mathematically expressed and incorporated within the overall optimization framework using a dynamic penalty approach. The key performance constraints addressed in the following sections include: the probability of power supply interruption (LPSP), capacity boundaries of individual system components, and the proportion of surplus or unused energy (EER)

### Loss of power supply probability (LPSP)

Reliability is a key performance indicator in isolated microgrids. The LPSP represents the fraction of time during which the system fails to meet demand and is expressed as:

24 $$\begin{aligned} \text {LPSP} = \frac{1}{8760} \sum _{t=1}^{8760} \Big [ P_{\text {load}}(t) -&\big ( P_{\text {solar}}(t) + P_{\text {wind}}(t) \nonumber \\&+ P_{\text {batt}}(t) + P_{\text {DG}}(t) \big ) \Big ]^+ \end{aligned}$$

where $[\cdot ]^+$ denotes the positive part operator, i.e., $\max (0, \cdot )$. A strict reliability condition is enforced by setting the maximum allowable LPSP to zero $(\beta _L = 0)$. Violations are penalized in the objective function.

### Component and system operating constraints

System components are constrained within their operational bounds to ensure feasibility and practical deployment. The constraints are defined as:

25 $$\begin{aligned} 0&< N_{\text {PV}} \le N_{\text {PV,max}} \end{aligned}$$

26 $$\begin{aligned} 0&< N_{\text {wind}} \le N_{\text {wind,max}} \end{aligned}$$

27 $$\begin{aligned} 0&< C_{\text {batt}} \le C_{\text {batt,max}} \end{aligned}$$

28 $$\begin{aligned} \text {SOC}_{\min }&\le \text {SOC}(t) \le \text {SOC}_{\max } \end{aligned}$$

29 $$\begin{aligned} P_{\text {DG,min}}&\le P_{\text {DG}}(t) \le \min (P_{\text {load,peak}}, P_{\text {DG,max}}) \end{aligned}$$

These ensure that sizing remains within acceptable technical specifications and that the dispatch strategy remains effective over time.

### Excess energy ratio (EER)

To quantify the surplus energy not consumed or stored, the Excess Energy Ratio (EER) is introduced:

30 $$\begin{aligned} \text {EER} = \frac{\sum _{t=1}^{8760} P_{\text {excess}}(t)}{\sum _{t=1}^{8760} P_{\text {total}}(t)} \end{aligned}$$

with:

31 $$\begin{aligned} P_{\text {excess}}(t) = \left[ P_{\text {solar}}(t) + P_{\text {wind}}(t) - P_{\text {batt}}(t) - P_{\text {load}}(t) \right] ^+ \cdot \Delta t \end{aligned}$$

Minimizing EER promotes better utilization of renewable generation and storage capacity, contributing to economic and environmental performance.To ensure feasible microgrid configurations, constraint violations are penalized within the objective function using the dynamic penalty formulation previously defined in Equation (16). This approach discourages solutions that exceed system limits (e.g., reliability thresholds, sizing bounds, or energy balance violations) by increasing their cost. By embedding penalties directly into the optimization process, the framework maintains both technical feasibility and economic efficiency throughout the search.

## Arctic Puffin Optimization (APO)

The Arctic Puffin Optimization (APO) algorithm is a recent metaheuristic inspired by puffins’ survival behaviors in both aerial and underwater environments. It proceeds through three stages: (i) initialization of the population, (ii) aerial flight for global exploration, and (iii) underwater foraging for intensive local refinement. This design enables a balance between diversification in early iterations and exploitation in later phases, which is particularly advantageous for complex multi-objective problems such as hybrid microgrid sizing.

### Behavior conversion factor B

The APO uses a transition coefficient *B* to achieve smooth transition between global exploration to local exploitation phases. This factor is defined as

32 $$\begin{aligned} B = 2 \times \log \!\left( \frac{1}{r}\right) \times \left( 1 - \frac{t'}{T_{\max }}\right) \end{aligned}$$

Where *r* is a random number between 0 and 1, $t'$ & $T_{\max }$ are the current and final iteration number respectively. *B* is usually compared to a value *C* (chosen by designer). If B is greater than *C*, the optimizer undergoes exploration, while if $\hat{B}$ becomes less than *C* the optimizer switches from the exploration to the exploitation phase. (*C*is set to 0.5).

### Population initialization

The search process begins by distributing the puffins (candidate solutions) uniformly across the feasible domain:

33 $$\begin{aligned} X_i^{t} = r\,(\textrm{ub} - \textrm{lb}) + \textrm{lb}, \qquad i = 1,2,\dots ,N \end{aligned}$$

where $X_i^{t}$ denotes the position of the *i*-th puffin at iteration *t*, $\textrm{lb}$ and $\textrm{ub}$ are the lower and upper bounds of the decision space, *r* is a uniformly distributed dimensionless random number in [0, 1], and *N* is the population size.

### Aerial flight stage (exploration)

After initialization, puffins perform aerial movements to diversify the search. This stage incorporates two complementary mechanisms: aerial search and swooping predation, which respectively allow wide-area exploration and intensified targeting of promising regions. The aerial search is expressed as:

34 $$\begin{aligned} \hat{Y}_i^{t+1} = \hat{X}_i^t + (\hat{X}_i^t - \hat{X}_r^t)\cdot L(D) + \hat{R} \end{aligned}$$

where $\hat{Y}_i^{t+1}$ is the updated position of puffin *i*, $\hat{X}_i^t$ is its previous position, $\hat{X}_r^t$ is a randomly selected puffin with $\hat{X}_r^t \ne \hat{X}_i^t$, *L*(*D*) is a Lévy-distributed step length in dimension *D*, and $\hat{R}$ is a corrective random step. The corrective step is defined as:

35 $$\begin{aligned} \hat{R} = \text {round}\left( 0.5\cdot (0.5+\hat{r})\right) \cdot \hat{\alpha } \end{aligned}$$

where $\hat{r}$ is a uniform random number in [0, 1] and $\hat{\alpha }$ is a normally distributed perturbation factor. Specifically,

36 $$\begin{aligned} \hat{\alpha } \sim \mathcal {N}(0,1) \end{aligned}$$

indicating that $\hat{\alpha }$ follows a standard Gaussian distribution with zero mean and unit variance.

The swooping predation step is modeled as:

37 $$\begin{aligned} \hat{Z}_i^{t+1} = \hat{Y}_i^{t+1}\cdot \hat{S} \end{aligned}$$

where $\hat{Z}_i^{t+1}$ is the updated dive position of puffin *i*, and $\hat{S}$ is a velocity coefficient controlling the dive intensity. This coefficient is computed as:

38 $$\begin{aligned} \hat{S} = \tan \!\left( (\hat{r}-0.5)\pi \right) \end{aligned}$$

where $\hat{r}$ is uniformly distributed in [0, 1].

The aerial population update is expressed as:

39 $$\begin{aligned} \hat{P}_i^{t+1} = \hat{Y}_i^{t+1} \cup \hat{Z}_i^{t+1} \end{aligned}$$

where $\hat{P}_i^{t+1}$ is the combined pool of puffin positions obtained from aerial search and swooping. Afterwards, sorting and selection are performed as:

40 $$\begin{aligned} \text {new} = \text {sort}(\hat{P}_i^{t+1}) \end{aligned}$$

41 $$\begin{aligned} \hat{X}_i^{t+1} = \text {new}(1:N) \end{aligned}$$

where $\text {sort}(\cdot )$ arranges candidates by fitness and $\hat{X}_i^{t+1}$ retains the best *N* puffins for the next iteration.

### Underwater foraging stage (exploitation)

Once potential regions are identified, puffins dive underwater to intensify the search. This stage comprises three sub-behaviors: cooperative gathering, intensification, and predator avoidance.

The cooperative gathering is expressed as:

42 $$\begin{aligned} \hat{W}_i^{t+1} = {\left\{ \begin{array}{ll} \hat{X}_i^t + F \cdot L(D)\cdot (\hat{X}_{r1}^t - \hat{X}_{r2}^t), & \text {if } \hat{r} \ge 0.5 \\ \hat{X}_i^t + F \cdot (\hat{X}_{r2}^t - \hat{X}_{r3}^t), & \text {if } \hat{r} < 0.5 \end{array}\right. } \end{aligned}$$

where $\hat{W}_i^{t+1}$ is the cooperative update of puffin *i*, *F* is the cooperation factor (set to 0.5 in this study), and $\hat{X}_{r1}^t, \hat{X}_{r2}^t, \hat{X}_{r3}^t$ are three distinct randomly chosen puffins.

An intensified search is carried out using:

43 $$\begin{aligned} \hat{Y}_i^{t+1} = \hat{W}_i^{t+1}\cdot (1+f) \end{aligned}$$

where f is an adaptive scaling parameter, computed as:

44 $$\begin{aligned} f = 0.1(\hat{r}-1)\cdot \frac{T_{\max }-t'}{T_{\max }} \end{aligned}$$

with $t'$ denoting the current iteration, $T_{\max }$ the maximum number of iterations, and $\hat{r}$ a random number in [0, 1].

Predator avoidance is modeled as:

45 $$\begin{aligned} \hat{Z}_i^{t+1} = {\left\{ \begin{array}{ll} \hat{X}_i^t + F \cdot L(D)\cdot (\hat{X}_{r1}^t - \hat{X}_{r2}^t), & \text {if } \hat{r} \ge 0.5 \\ \hat{X}_i^t + \hat{\beta } \cdot (\hat{X}_{r2}^t - \hat{X}_{r3}^t), & \text {if } \hat{r} < 0.5 \end{array}\right. } \end{aligned}$$

where $\hat{Z}_i^{t+1}$ is the avoidance position of puffin *i* and $\hat{\beta }$ is a random coefficient drawn uniformly from [0, 1].

**Fig. 2:**
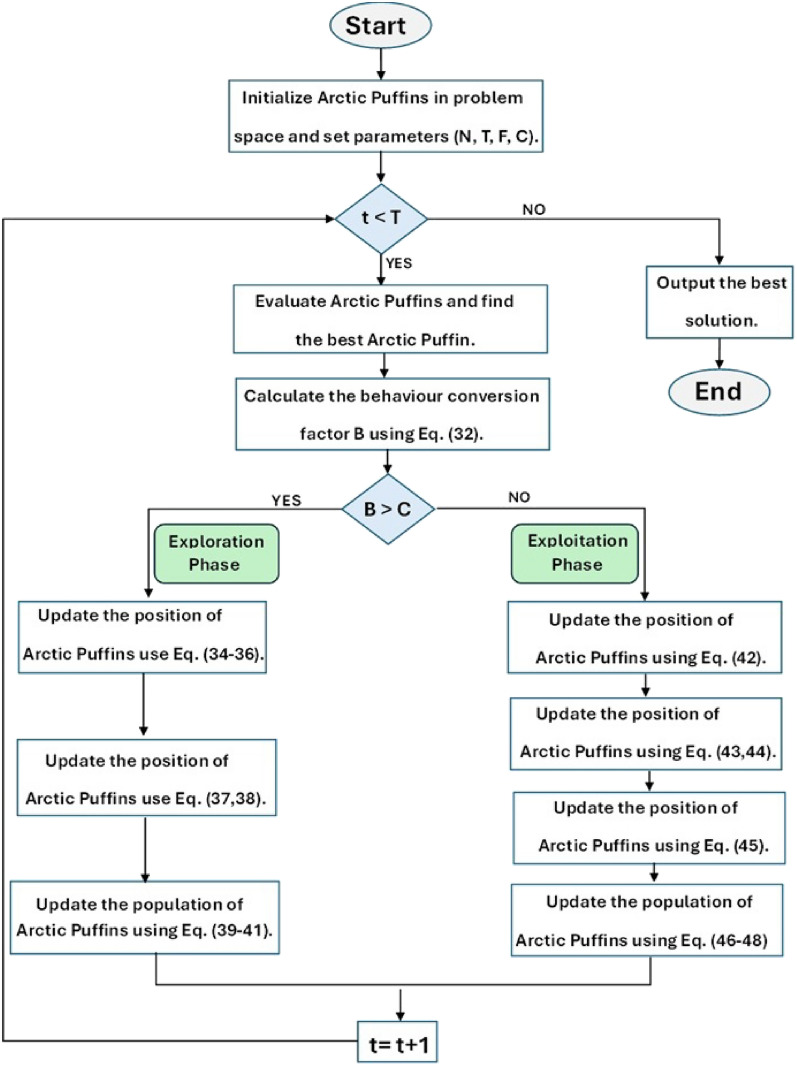
APO flow chart.

The underwater update process then combines all three strategies:

46 $$\begin{aligned} \hat{P}_i^{t+1} = \hat{W}_i^{t+1} \cup \hat{Y}_i^{t+1} \cup \hat{Z}_i^{t+1} \end{aligned}$$

47 $$\begin{aligned} \text {new} = \text {sort}(\hat{P}_i^{t+1}) \end{aligned}$$

48 $$\begin{aligned} \hat{X}_i^{t+1} = \text {new}(1:N) \end{aligned}$$

where the combined candidate pool $\hat{P}_i^{t+1}$ is sorted by fitness and truncated to retain the top *N* puffins. The integration of aerial exploration and underwater exploitation forms the complete APO cycle. A schematic overview is provided in Fig. 2, while the parameter settings adopted for all simulation are summarized in Table 4. The values were selected through preliminary tuning and corroborated by related studies to ensure fairness and reproducibility.

**Table 4:** APO Algorithm Parameters.

Parameter	Value
Population size	50
Maximum iterations	50
Cost weight in objective	0.6 (assumed)
Emissions weight in objective	0.4 (assumed)
Penalty coefficient	1000 (adaptive)
Random coefficients	Uniform in [0, 1]
Stochastic term	Gaussian (mean 0, std 1)

## Simulation setup and results

This section describes the simulation setup employed to assess the effectiveness of the Arctic Puffin Optimization (APO) algorithm for hybrid microgrid planning. It outlines the test scenarios, key simulation settings, and validation approach used to compare APO with other advanced metaheuristic techniques.

### Simulation validation and case study design

To evaluate the performance of the proposed Arctic Puffin Optimization (APO) algorithm under realistic operational conditions, an experimental study was conducted using actual meteorological and demand data. The APO algorithm was benchmarked against three widely recognized metaheuristics–Grey Wolf Optimizer (GWO), Ant Lion Optimizer (ALO), and Starfish Optimization Algorithm (SFOA)–all of which have demonstrated effectiveness in solving multi-objective, constrained optimization problems in energy systems.Two case scenarios were developed based on data from Ras Ghareb, Egypt–a coastal region with strong wind resources and high solar irradiance. In Scenario 1, the isolated microgrid was configured with three elements: WT, DG, and BESS.. The second scenario built upon this configuration by integrating photovoltaic (PV) panels to increase renewable energy share and reduce reliance on diesel fuel. To capture seasonal variability, two representative load profiles were used: one reflecting summer demand and the other representing winter consumption. These profiles were derived from normalized daily residential and commercial data and scaled to an annual horizon, allowing comprehensive performance analysis across peak and off-peak seasons.

To ensure a fair comparison, all metaheuristic algorithms (APO, GWO, ALO, and SFOA) were executed using identical population sizes (50 agents) and the same maximum number of iterations (50), following the standard practice in benchmarking studies. The control parameters for each algorithm were adopted directly from their original formulations without additional tuning or bias in favor of any method. This standardization ensures that the performance differences observed in the results arise from the intrinsic search mechanisms of each algorithm rather than unequal or optimized parameter settings.

System configurations were assessed using four main performance metrics: annual system cost, CO$_2$ emissions, the probability of power supply shortfalls, and the ratio of surplus energy that exceeds demand. These metrics collectively assess economic feasibility, environmental impact, energy reliability, and operational efficiency. All simulations were performed on a workstation equipped with an Intel Core i7 processor (3.2 GHz), 16 GB RAM, running Windows 10 and MATLAB R2023a . For each candidate microgrid configuration, the techno-economic model is evaluated at an hourly time step over a full annual horizon (8,760 samples), using deterministic time series for solar irradiance, wind speed, and load demand. The APO, GWO, ALO, and SFOA algorithms are implemented in MATLAB as population-based solvers with 50 search agents and 50 iterations per run. The objective function and constraints are computed directly via custom m-files without calling external linear, mixed-integer, or nonlinear programming solvers; thus, the metaheuristic engines themselves act as the optimization solvers. This setup provides a transparent and reproducible simulation environment with a clearly defined sampling resolution and algorithmic configuration.

.The APO algorithm was adapted from the original formulation proposed by Wang et al.^48^ and its reference implementation on MATLAB Central File Exchange^49^. Comparator algorithms, including GWO, ALO, and SFOA, were implemented based on standard formulations . All simulations were conducted without the use of external toolboxes to ensure transparency and reproducibility. Simulation results were validated against benchmark studies in the literature to confirm model accuracy.Constraint violations, including unmet demand, system oversizing, and energy overproduction, were penalized dynamically within the objective function. This adaptive penalty mechanism steered the optimization process toward feasible and high-performing microgrid designs.

To ensure efficient parameter-space exploration and reliable convergence, the optimization process was designed around a balanced exploration–exploitation strategy. The search begins with a global exploration phase, where wide and diverse candidate updates are generated to cover the full range of feasible system configurations and avoid premature convergence. As the search progresses, the algorithm gradually shifts toward a more focused exploitation phase, refining promising solutions through localized adjustments and cooperative interactions among candidate solutions. Infeasible designs–such as those resulting in unmet demand or excessive excess energy–are automatically discouraged through penalty-based evaluations, which guide the search away from unrealistic or impractical regions of the solution space. All optimization runs were performed using identical population sizes and iteration counts across all compared algorithms, ensuring fairness and consistency. Multiple independent runs were also conducted to verify repeatability, and the resulting trends confirm that the proposed method converges efficiently and consistently toward high-quality solutions.

In this work, the annual time series used for solar irradiance, wind speed, and load demand are treated as a fixed benchmark dataset that is applied uniformly to all optimization runs. By evaluating APO, GWO, ALO, and SFOA under exactly the same hourly boundary conditions, the observed differences in convergence behaviour and final solutions can be attributed to the intrinsic search dynamics of each algorithm rather than to stochastic variations in the input data. Seasonal variability at the Ras Ghareb site is still fully represented through the chosen historical year, while the impact of broader resource and demand uncertainties is examined separately via the parametric sensitivity analysis presented in the following subsection.

### Simulation and results

**Fig. 3:**
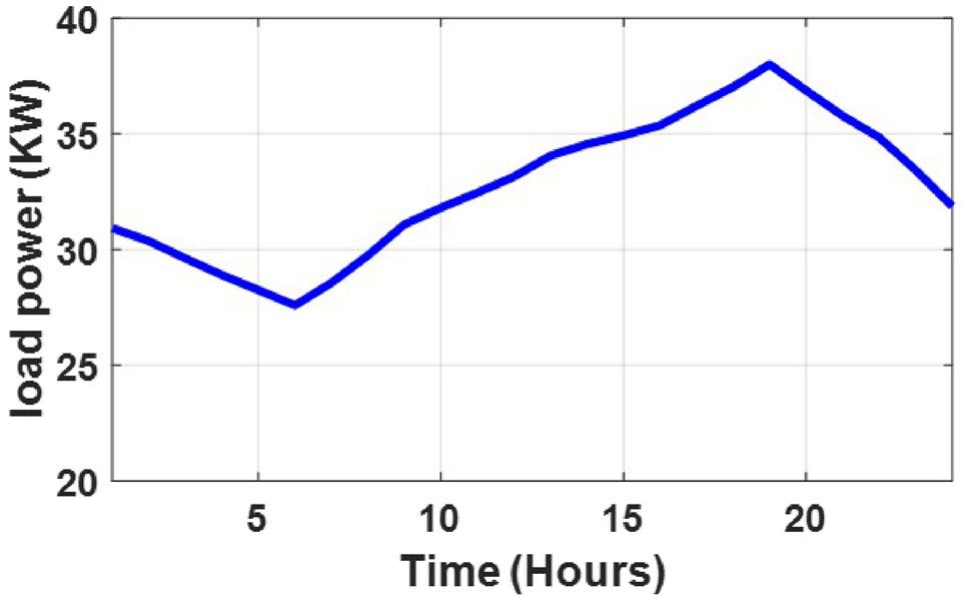
Load demand in winter day.

**Fig. 4:**
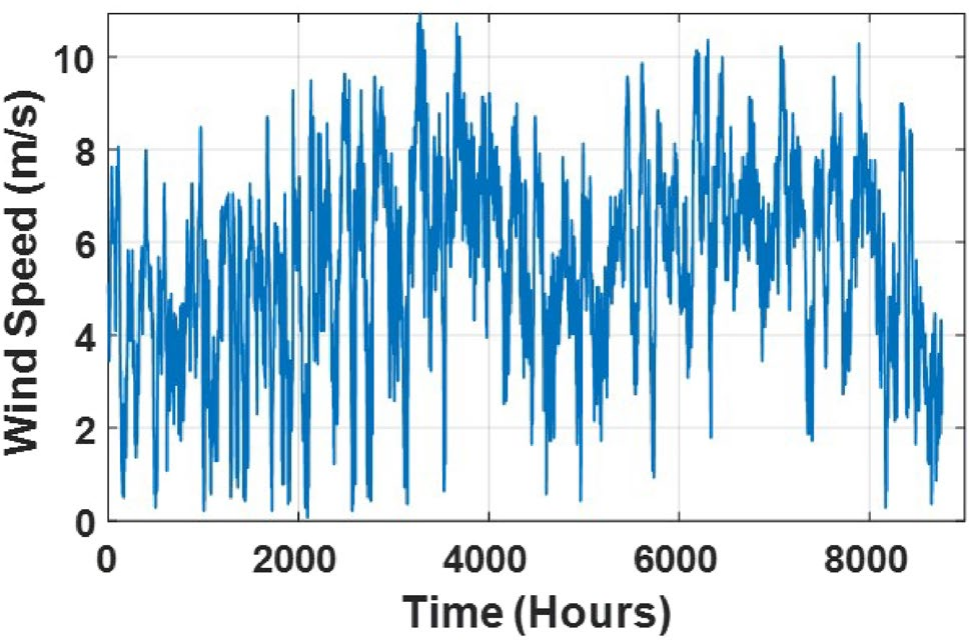
Wind speed in a year.

**Fig. 5:**
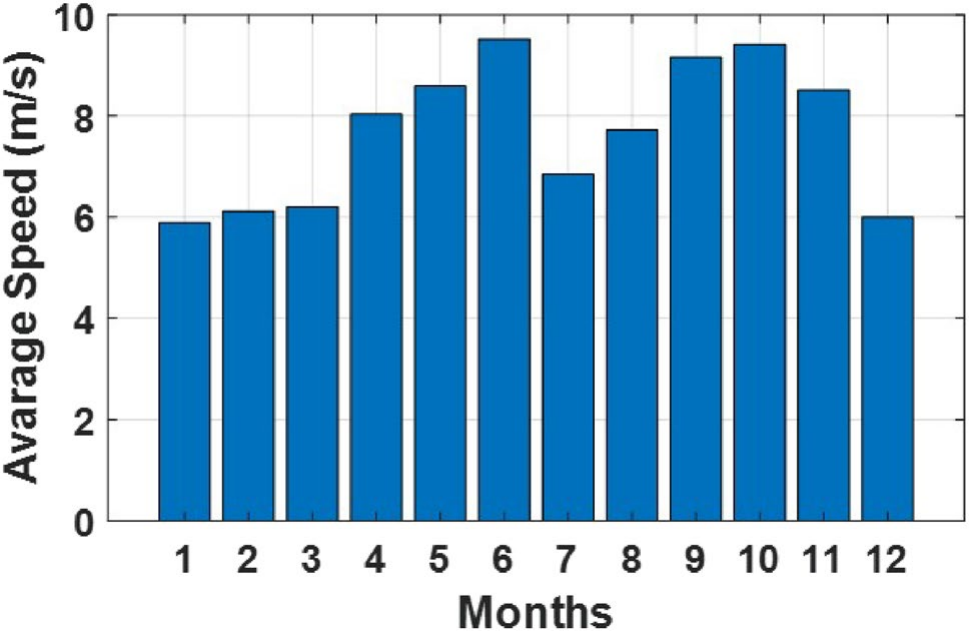
Average wind speed in a month.

**Fig. 6:**
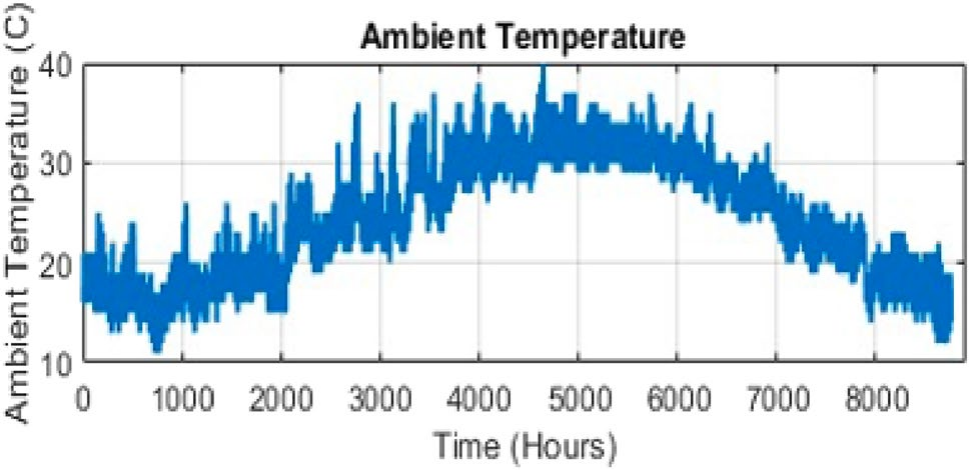
Ambient temperature in year.

**Fig. 7:**
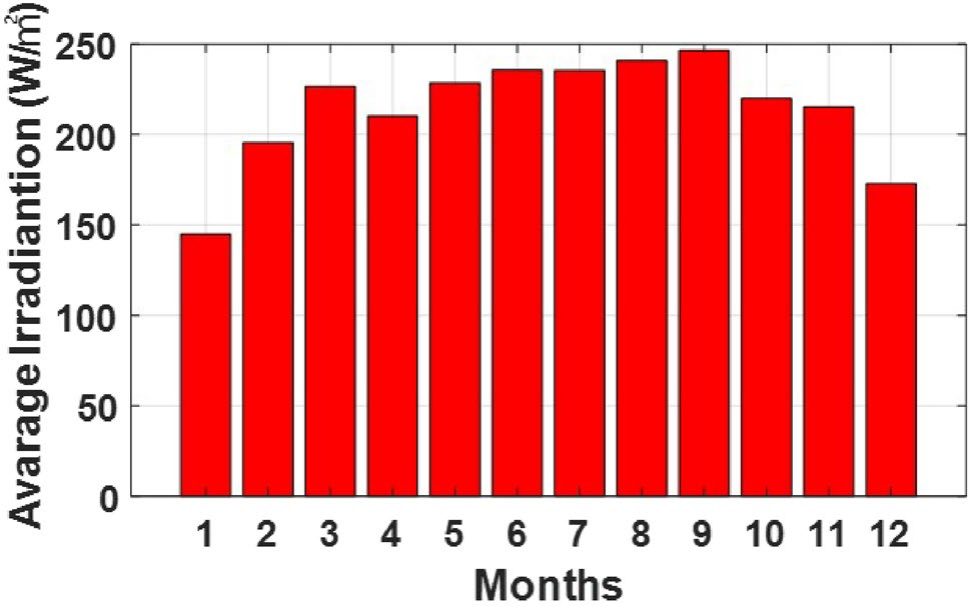
Monthly average irradiance.

**Fig. 8:**
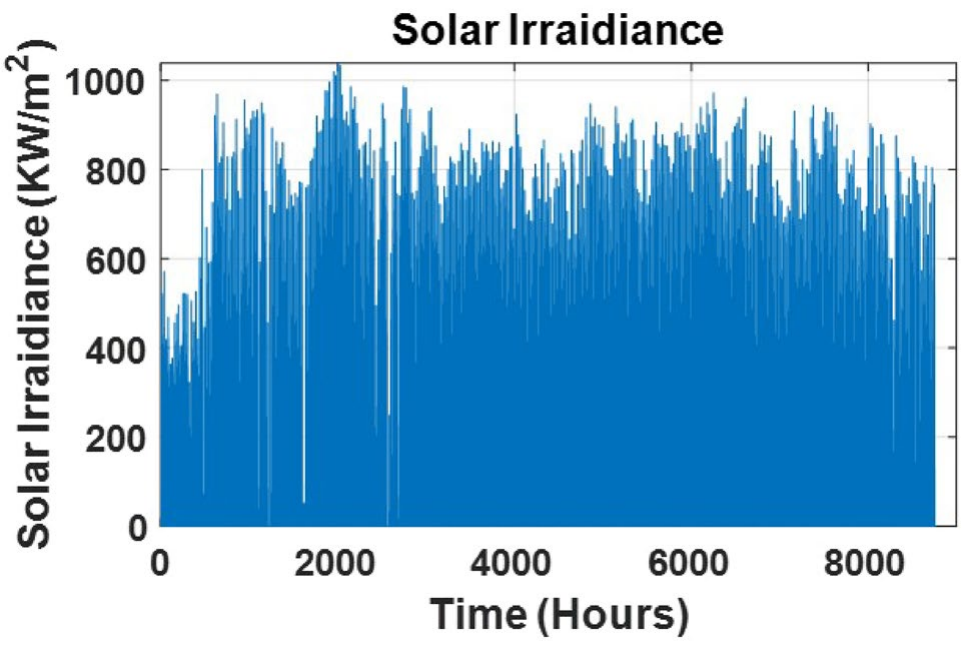
Irradiance in a year.

The system analysis begins with the load demand profiles across seasonal conditions. Figure 3 illustrates the summer day load, which peaks around 850 W at 14:00 and has a secondary peak of 780 W near 21:00. Morning demand starts at approximately 400 W and rises steeply by midday, reflecting high cooling loads during hot hours and aligning well with solar PV output potential. In contrast, Fig. 4 shows the winter day load, where the peak occurs around 19:00 at 650 W, with a noticeable morning rise to 600 W by 08:00, likely due to heating and lighting needs. Daytime loads are more stable, averaging around 500 W, making them more suitable for wind and diesel generator (DG) coverage. Figures 4 and 5 reveal how wind resources vary across the year. Wind speeds fluctuate widely, sometimes exceeding ${12} \hbox { m s}^{-1}$, but often falling below ${3}\hbox { m s}^{-1}$, which is the minimum required to trigger turbine operation. This irregularity becomes more noticeable in the summer, when long stretches of low-speed wind reduce the effectiveness of wind energy harvesting. Monthly averages shown in Fig. 5 confirm that March and November tend to be the strongest wind months, with speeds near ${7.5}\hbox { m s}^{-1}$, while August and September register the weakest performance at about ${5.5}\hbox { m s}^{-1}$. Figure 6 highlights how ambient temperatures shift throughout the year, starting from about ${13}^{\circ }\hbox {C}$ in winter and climbing to over ${40}^{\circ }\hbox {C}$ in peak summer. These high temperatures, particularly between June and August, can reduce PV efficiency due to heat-induced losses. Meanwhile, solar resource availability is shown in Figs. 7 and 8. The data indicate that monthly average irradiance exceeds ${850} \hbox { W m}^{2}$ in June and remains above ${700} \hbox { W m}^{2}$ throughout the summer, providing excellent conditions for solar power production. The annual profile in Fig. 8 follows the same pattern, with strong mid-year irradiance and weaker levels in winter.

**Fig. 9:**
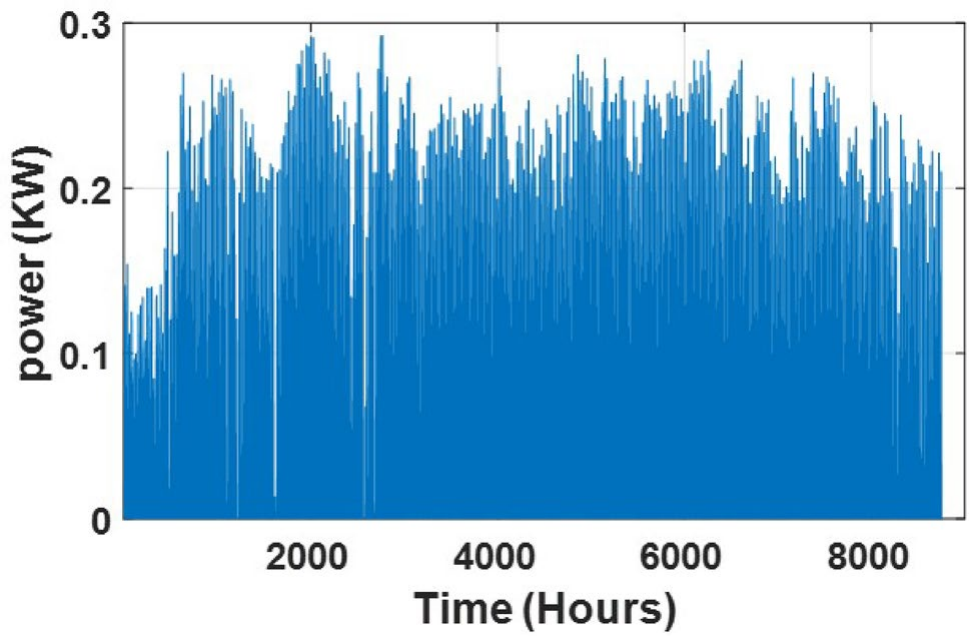
Solar power output in a year.

Figure 9 reflects how these environmental patterns translate into actual PV output. During summer, solar generation frequently surpasses 1000 W, ensuring high daytime energy yield. In contrast, winter months show reduced output, sometimes dropping below 400 W, which increases reliance on diesel generation or battery storage.

**Fig. 10:**
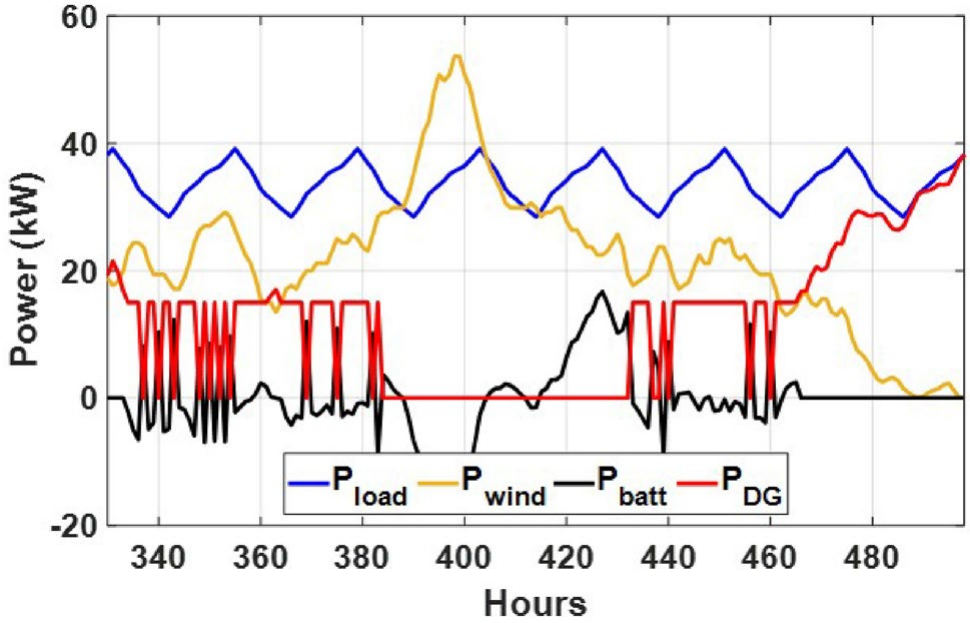
Summer week power flow distribution for Case 1 showing load, wind, DG, and battery power (kW) over time (h).

**Fig. 11:**
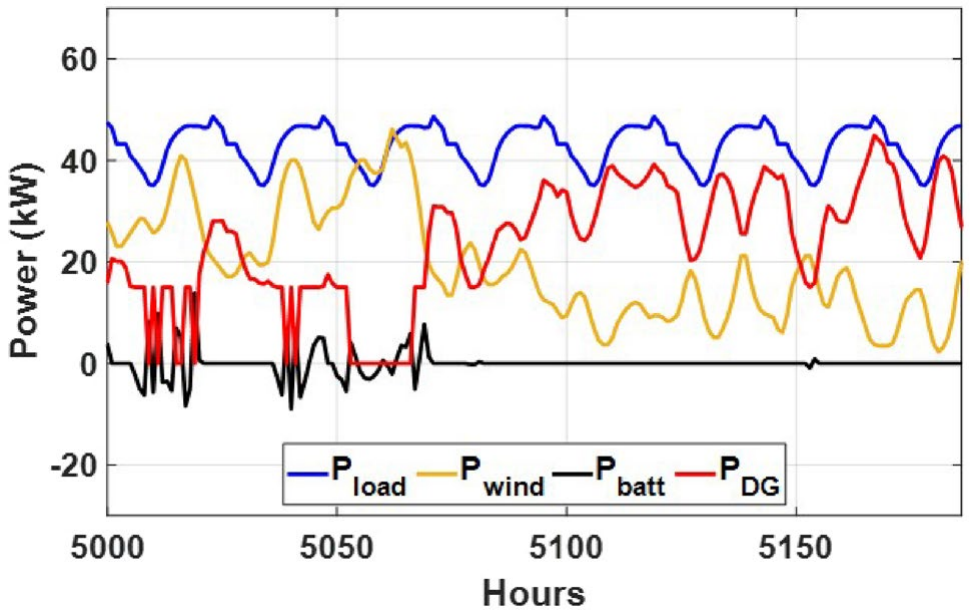
Winter week power flow distribution for Case 1 showing load, wind, DG, and battery power (kW) over time (h).

**Fig. 12:**
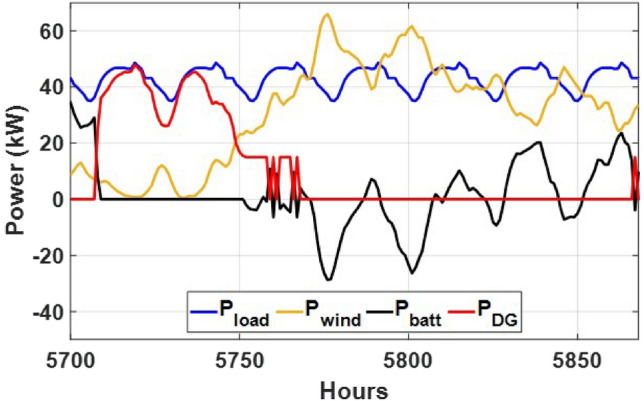
Autumn week power flow distribution for Case 1 with load, wind, DG, and battery power shown in kW over time (h).

**Fig. 13:**
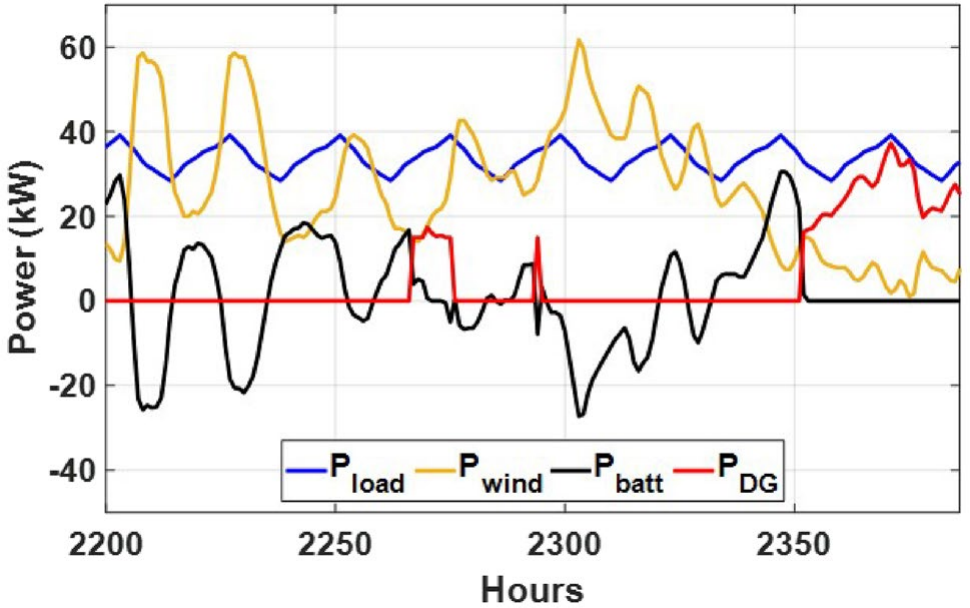
Spring week power flow distribution for Case 1 illustrating load, wind, DG, and battery power (kW) versus time (h).

**Fig. 14:**
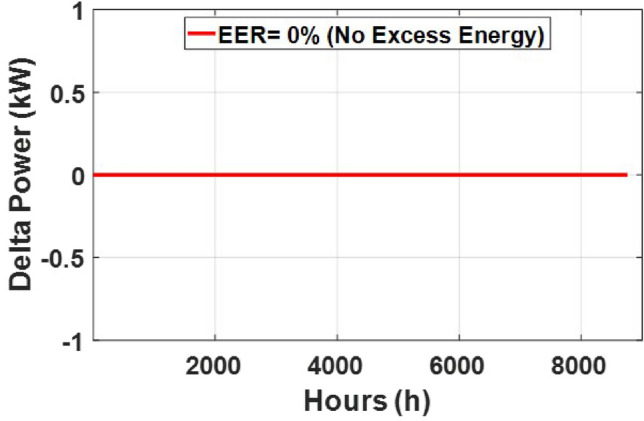
Excess energy behaviour (EER) for Case 1 shown in kW/kWh versus time (h).

**Fig. 15:**
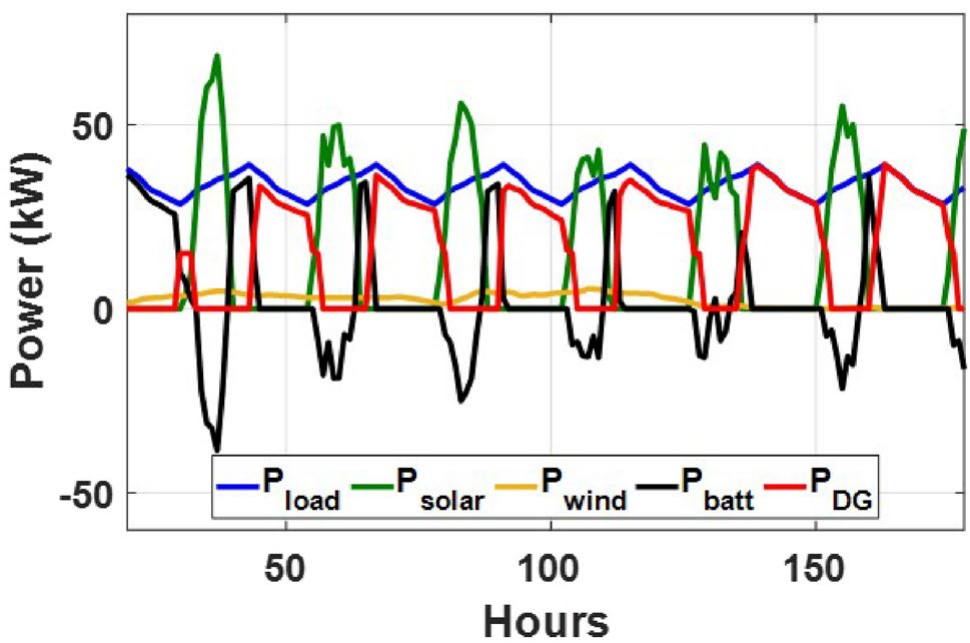
Winter week power flow distribution for Case 2 with PV, wind, DG, and battery power (kW) over time (h).

**Fig. 16:**
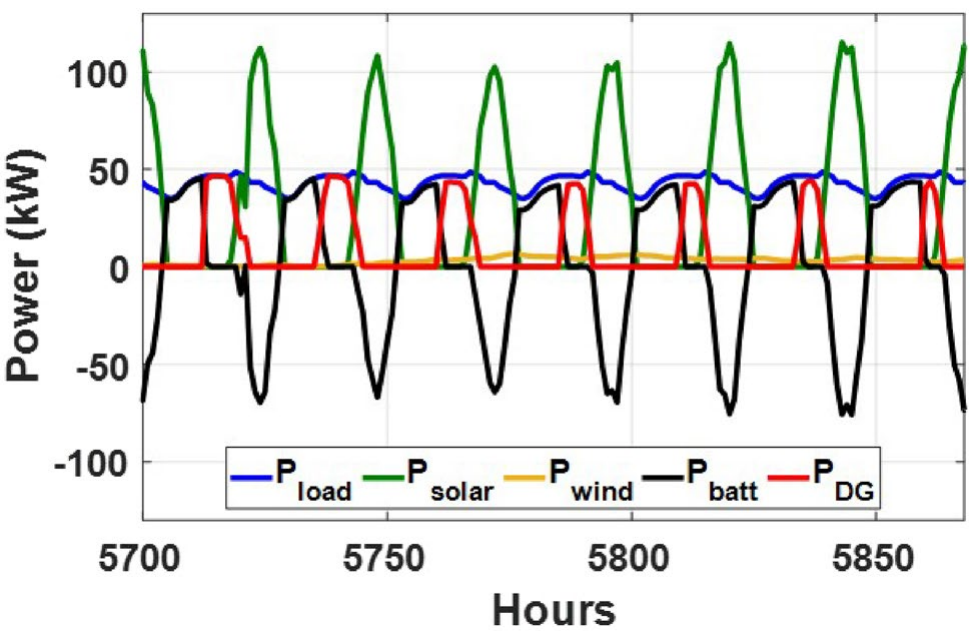
Autumn week power flow distribution for Case 2 showing PV, wind, DG, and battery power (kW) versus time (h).

**Fig. 17:**
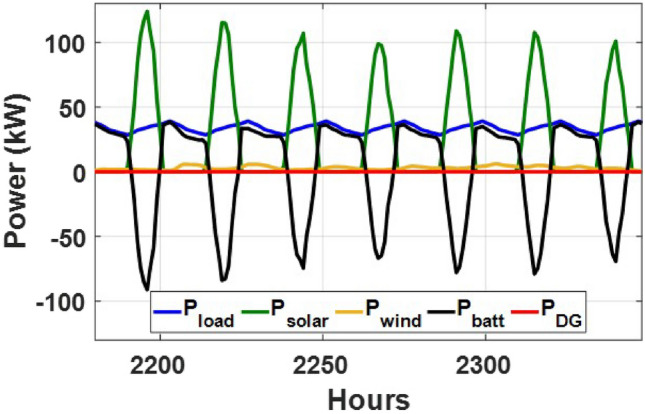
Spring week power flow distribution for Case 2 with PV, wind, DG, and battery power shown in kW over time (h).

**Fig. 18:**
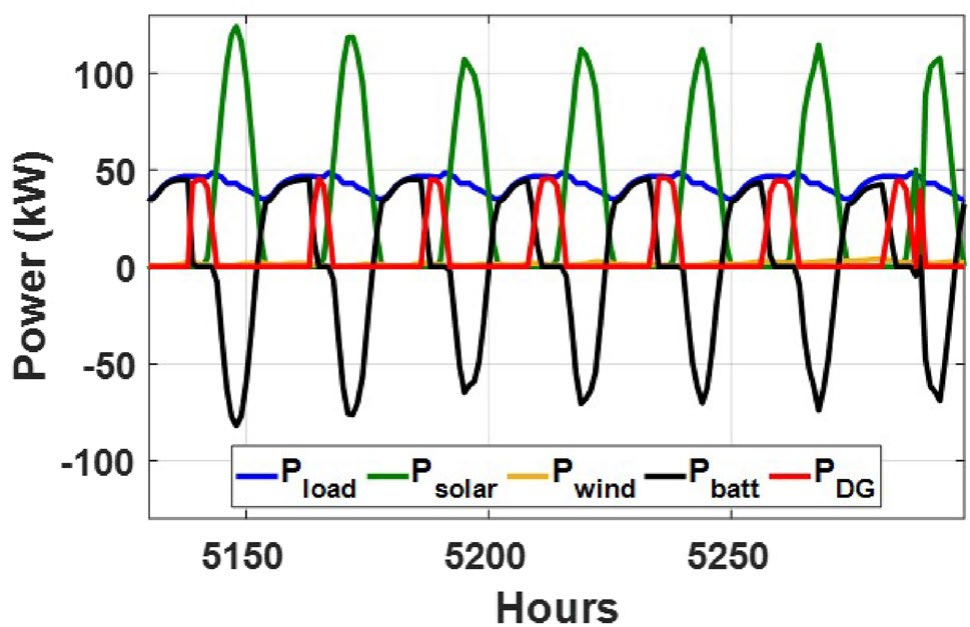
Summer week power flow distribution for Case 2 illustrating PV, wind, DG, and battery power (kW) versus time (h).

These seasonal trends highlight the complementary behavior of solar and wind, supporting the case for a hybrid configuration over single-source setups.The seasonal power balance profiles illustrated in Figs. 10, 11, 12, 13 and 14 provide a comprehensive validation of the proposed APO-based optimization framework for the isolated hybrid microgrid composed of wind turbines, diesel generator (DG), and battery storage. In winter (Fig. 10), wind energy dominates the supply, covering approximately 85% of the weekly demand, while the DG and battery support load balancing during wind troughs. Summer operation (Fig. 11) reflects increased energy demand and variability, resulting in a higher reliance on the DG, which contributes up to 30% of the load, with batteries actively compensating during low-wind periods. Autumn conditions (Fig. 12) show a well-balanced contribution from wind and storage, with DG usage limited to brief intervals, achieving a renewable fraction of over 90%. Spring operation (Fig. 13) showcases near-optimal renewable penetration, where wind energy alone meets nearly 95% of the load, and both DG and battery remain minimally engaged. Most notably, Fig. 14 confirms that the system consistently operates without producing excess energy, indicating an Excess Energy Ratio (EER) of zero. This confirms that the APO algorithm enables high renewable penetration, accurate component sizing, and strong seasonal adaptability–key requirements for cost-effective and practical deployment in off-grid microgrids.Table 5 presents the optimization results of the applied algorithms (GWO, ALO, SFOA, and APO).As for Case Study 2, the following results illustrate the system’s behavior across different conditions. Figures 15,16,17 and 18 illustrate the power balance across different seasonal weeks– winter, autumn,spring , and summer, respectively. When viewed alongside earlier optimisation studies on hybrid PV–WT–DG–battery systems, the results of this work highlight a broader methodological advancement rather than a numerical comparison alone. Many prior approaches optimise sizing and dispatch separately, rely on simplified resource assumptions, or exclude degradation and emissions from the objective function. In contrast, the present framework integrates these aspects into a unified multi-objective formulation and evaluates system performance using full-year time-series data. As a result, the improvements observed with APO reflect not only the search capability of the algorithm but also the advantages of employing a more comprehensive optimisation structure than those commonly used in previous literature.

**Fig. 19:**
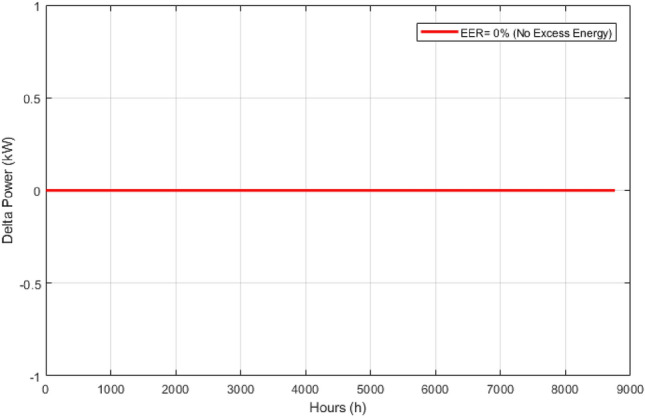
Excess energy behavior (EER) for Case 2 shown in kW/kWh versus time (h).

These results demonstrate the system’s capability to effectively blend multiple energy sources, adapting seamlessly throughout the year. During periods of high sunlight, PV generation takes the lead, while wind energy steps in during times of lower irradiance, creating a balanced dynamic. Meanwhile, the diesel generator and battery storage serve as critical backups, ensuring consistent reliability and uninterrupted power availability. Figure 19 shows that the system operates without producing excess energy, indicating efficient sizing and energy management across the hybrid components.

Beyond solution quality, it is important to consider the practical implications of computational effort when applying metaheuristic solvers to long-term microgrid planning. In this study, all algorithms operate under the same population size and iteration budget, ensuring that runtime differences arise solely from their internal search dynamics. Although APO involves a richer two-stage update mechanism, its runtime remains well within acceptable limits for offline techno-economic planning, where solutions are computed hours or days before deployment rather than in real time. Thus, the computational effort should be interpreted in the context of planning applications, where marginal increases in runtime are typically justified by improvements in solution robustness and cost–emission performance.

In the microgrid literature, the excess energy profile is widely recognized as a key indicator of system sizing accuracy, renewable utilization efficiency, and operational stability. Studies on PV–wind–DG–battery systems highlight that high excess energy typically indicates oversized renewable capacity or inadequate dispatch coordination, which leads to curtailment and economic inefficiencies. Conversely, the near-zero Excess Energy Ratio (EER) observed in Case 2 is widely interpreted as evidence of balanced sizing and effective energy management, ensuring that available renewable generation is fully utilized. This behavior is consistent with findings in previous hybrid microgrid studies, where minimal excess energy is associated with improved economic performance and enhanced system reliability.

**Fig. 20:**
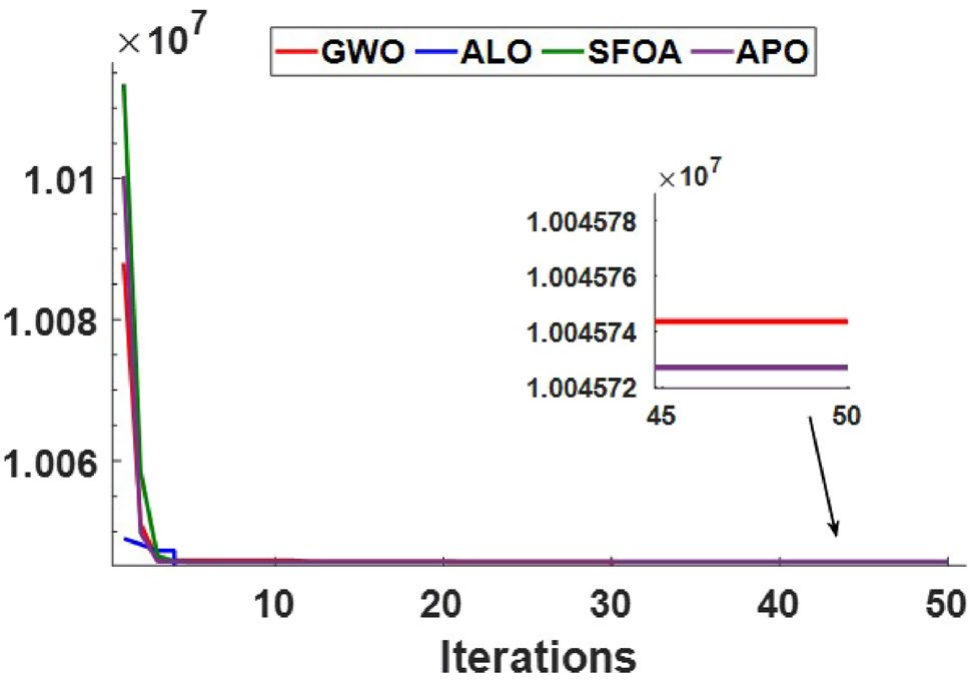
Convergence curves of APO, GWO, ALO, and SFOA over 50 iterations, showing objective value versus iteration.

Meanwhile, Fig. 20 presents the convergence behavior of the tested algorithms, where the Arctic Puffin Optimization (APO) demonstrates faster convergence and superior performance compared to GWO, ALO, and SFOA. These results highlight the robustness of APO not only in minimizing costs and emissions but also in achieving rapid and stable optimization under varying operational conditions.

Based on these comparative tests, the APO algorithm demonstrates several clear strengths relative to GWO, ALO, and SFOA. APO consistently achieves lower annual system cost, higher renewable penetration, and maintains LPSP = 0 and a near-zero EER across all scenarios, reflecting superior sizing accuracy and more effective dispatch coordination. The convergence trends further show that APO reaches high-quality solutions more rapidly and with greater stability. These strengths stem from APO’s balanced exploration–exploitation design and its two-stage search mechanism. However, the results also highlight APO’s main limitation: its moderately higher computational runtime compared to the benchmark algorithms, due to the increased number of candidate evaluations per iteration. Thus, while APO offers higher solution quality and robustness, its computational overhead should be considered when scaling to larger systems or real-time control applications.

Table 6 summarizes the optimization results for the proposed hybrid microgrid.The results indicate that the APO algorithm significantly outperforms the others, providing the lowest annual system cost–approximately 4% to 8% lower compared to the other algorithms–along. Additionally, the renewable energy fraction achieved by APO is notably higher by around 15% to 17%, underscoring its effectiveness in maximizing the use of renewable energy sources compared to GWO, ALO, and SFOA. From a computational standpoint, APO requires a longer runtime per execution compared to GWO, ALO, and SFOA, as reported in Tables 5 and 6. For Case 1, the average time per run is approximately 15–16 s for APO versus 4–11 s for the other algorithms, while in Case 2 APO requires about 13 s per run compared to 3–6 s for the benchmarks. This overhead is mainly due to the two-stage search process (aerial exploration and underwater exploitation) and the use of Lévy-flight-based moves, which increase the number of candidate updates per iteration. Nevertheless, the per-iteration computational complexity of APO remains on the same order as other population-based metaheuristics, i.e., $\mathcal {O}(N \cdot D)$, where *N* is the population size and *D* is the number of decision variables.

In addition to the comparison with GWO, ALO, and SFOA, the obtained APO results were also examined against general performance ranges reported in earlier optimisation studies on PV–WT–DG–BESS systems. Prior works using PSO, GA, HHO, MILP-based approaches, and various hybrid metaheuristics typically report renewable fractions in the range of 55–70%, higher annual system costs, and, in some cases, non-zero LPSP when evaluated under realistic meteorological conditions. In contrast, the APO-based configuration in this study achieves a higher renewable share together with lower annual cost while maintaining LPSP = 0 across all scenarios, indicating that the optimisation results are consistent with or superior to the typical performance ranges documented in the literature.

**Table 5:** Case Study 1: (WT/DG/BESS) key findings.

Metric	GWO	ALO	SFOA	APO
MIN. ASC ($)	589952.73	602678.33	595927.36	**588872.57**
Inc. (%)	0.183	2.3	1.198	**0**
AVG. ASC ($)	9667499.39	9679937.25	7252003.98	**7042869.73**
STAND. DEV.	1871918.66	1811364.30	4311396.98	**4464757.05**
AVG. TIME PER RUN (s)	5.79	10.97	4.46	**15.67**
$$N_{\text {wind}}$$	82	81	82	**81**
$$C_{\text {BATT}}$$ [Wh]	4558200	4671600	4611600	**4548900**
DG_RATINGS [kW]	48.0064	48.0144	48.0064	**48.0144**
RF (%)	71.52	71.20	71.54	**70.23**
EER (%)	0.0	0.0	0.0	**0.0**

**Table 6:** Case Study 2: (PV/WT/DG/BESS) key findings.

Metric	GWO	ALO	SFOA	APO
MIN. ASC ($)	149921.55	183267.93	149887.79	**141247.20**
Inc. (%)	6.14	26.28	6.11	**0**
AVG. ASC ($)	5942952.96	9848727.25	9548950.98	**8090233.97**
STAND. DEV.	4972545.64	1394799.56	4772730.08	**4006782.21**
AVG. TIME PER RUN (s)	3.83	6.28	3.75	**13.19**
$$N_{\text {pv}}$$	454	435	439	**446**
$$N_{\text {wind}}$$	7	12	9	**8**
$$C_{\text {BATT}}$$ [Wh]	630000	935100	612000	**541800**
DG_RATINGS [kW]	46.6437	47.7062	47.9446	**48.0241**
RF (%)	74.63	73.85	70.14	**70.37**
EER (%)	0.0	0.0	0.0	**0.0**

### Sensitivity analysis

In this study, the sensitivity analysis follows a one-factor-at-a-time (OFAT) approach in which a single variable is perturbed while all others remain fixed at their APO-optimized baseline values. Load demand, wind speed, solar irradiance, and diesel generator loading were independently varied within a range of $\pm 20\%$ to $25\%$ using 5% increments. For each perturbed case, the APO optimisation was repeated over the full annual time-series (8,760 hours), and the resulting annual system cost, fuel and emission components, renewable fraction, and LPSP were quantified. This structured procedure isolates the influence of each parameter and enables a systematic evaluation of how individual uncertainties propagate through the techno-economic performance of the microgrid.

A comprehensive sensitivity analysis was conducted for both Case Study 1 and Case Study 2 to evaluate the impact of operational variations on the economic and environmental performance of hybrid microgrid systems. In the first case study, which involves wind turbines, a diesel generator, and battery storage, the load demand was incrementally raised to 125% in 5% intervals (as shown in Table 7). This escalation resulted in increased annual system costs, greater fuel usage, and higher emissions, primarily due to the more frequent operation of the diesel generator. Similarly, reducing DG loading from 100% to 75% (Table 8) resulted in decreased efficiency and elevated system costs. Variations in wind speed also played a significant role: reduced wind speed by up to 20% (Table 9) increased reliance on DG, while increasing wind speed by the same percentage (Table 10) reduced both ASC and emissions, demonstrating the benefits of enhanced renewable input. In the second case study, which incorporates solar panels alongside wind turbines, a diesel generator, and battery storage, the integration of photovoltaic generation enhanced system flexibility and improved overall resilience.Table 11 presents the sensitivity analysis on load demand, where the system’s response is evaluated under incremental increases from 100% to 125%. As demand rises, the annualized system cost (ASC) increases consistently, reaching a 19.65% growth at 125% load. This is primarily due to a substantial rise in fuel and emission costs, stemming from greater dependence on the diesel generator. The renewable fraction (RF) declines from 70.37% to 37.91%, signaling a reduced contribution from renewable sources. Despite these degradations, the loss of power supply probability (LPSP) remains at 0%, indicating that system reliability is preserved, albeit at the cost of sustainability. These findings suggest that the current renewable configuration faces scalability limitations under rising load conditions. Table 12 investigates the effect of reducing the rated power of the diesel generator in 5% steps down to 75% of its original size. The results reveal a relatively minor impact on performance, with ASC rising by only 4.48%. Fuel and emission costs increase modestly, while RF remains largely stable around 70.37%, and LPSP continues at 0%. This indicates that the system is robust to reductions in DG capacity and can maintain supply reliability with only minor economic penalties–making downsizing a viable option for cost-saving or environmental objectives. Table 13 explores the system’s sensitivity to decreasing wind speed (up to a 25% reduction). The ASC shows only a slight increase of 2.59%, accompanied by gradual rises in fuel and emission costs. The RF decreases modestly to 64.57%, but LPSP remains at 0% throughout. These outcomes highlight the system’s moderate reliance on wind energy, which, while beneficial, is not a critical component for maintaining supply security under current sizing. Conversely, Table 14 evaluates wind power enhancement up to 125% of baseline. This scenario leads to a 2.81% reduction in ASC, accompanied by notable drops in fuel and emission costs. The RF improves to 76.31%, demonstrating the value of increased wind penetration. However, an anomalous LPSP value of -1% appears, likely due to a reporting or rounding artifact or potential over-generation not properly accounted for–possibly indicating curtailed or unutilized energy.

It is important to note that the negative LPSP values reported in Table 14 do not represent an actual loss-of-power condition. This anomaly arises from numerical rounding during post-processing when the calculated unmet-load term becomes slightly negative due to floating-point precision limits and the presence of surplus renewable generation. Because LPSP is theoretically bounded between 0 and 1, any negative value is physically meaningless and should be interpreted as zero. The corrected LPSP value for these cases is therefore 0, indicating that the system maintains full reliability even under enhanced wind conditions. Importantly, this numerical artifact has no impact on the optimization results or the conclusions drawn from the sensitivity analysis.

Lastly, Table 15 examines reductions in PV capacity, showing that the system is highly sensitive to solar resource availability. A 25% reduction in irradiance results in a sharp 14.28% increase in ASC, along with significant increases in fuel and emission costs. RF declines precipitously to 30.37%, though LPSP remains at 0%. This underscores the dominant role of solar PV in the hybrid system architecture. The findings emphasize that reductions in PV output disproportionately burden the diesel generator, thereby compromising the cost-effectiveness and environmental integrity of the system. Finally, Table 16 compares different generation configurations–Wind only, PV and Wind, and DG only–and confirms that the combined use of PV and Wind delivers the lowest ASC and emissions, establishing it as the most sustainable and cost-effective option among the scenarios analyzed. The simulation and sensitivity analysis confirm the effectiveness of the APO algorithm in minimizing system cost, emissions, and fuel use across varying seasonal conditions. APO consistently outperforms other metaheuristics while maintaining zero Loss of Power Supply Probability (LPSP), demonstrating strong potential for off-grid applications like Ras Ghareb. Wind and solar availability emerged as the most influential factors, emphasizing the need for precise resource assessment.

Although the optimisation framework relies on deterministic historical profiles, the extensive OFAT sensitivity experiments implicitly account for typical uncertainty ranges in renewable resources and demand. The consistent trends observed under $\pm 20\%$ perturbations confirm that the APO-optimised configuration is resilient to normal year-to-year variability. Therefore, even without explicit stochastic modelling, the combined deterministic simulation and sensitivity analysis provide strong evidence that the main conclusions regarding cost, reliability, and renewable utilisation remain valid under realistic uncertainty conditions.

The proposed PV–WT–DG–BESS microgrid configuration is practically feasible and aligned with commercially available technologies. Each subsystem—photovoltaic arrays, small-scale wind turbines, diesel generators, and lithium-ion battery banks—is widely deployed in remote and off-grid regions, including Egypt. The control strategy relies on standard inverter–battery management functions used in existing hybrid systems, and all components operate within rated limits validated in the literature. The APO-based planning framework is applied offline, meaning that its computational complexity does not affect real-time operation, and the resulting system sizes can be implemented using current microgrid hardware. Therefore, despite the apparent complexity of the optimization framework, the final engineered system is fully compatible with practical field deployment and existing microgrid standards.

Before concluding, it is important to highlight that the feasibility results reinforce the practical deployability of the optimized configuration, strengthening the validity of the APO-based design for real-world applications.

Overall, the sensitivity findings confirm that the APO-based microgrid design remains stable across all tested variations, with LPSP consistently equal to zero and no abrupt degradation in economic or renewable performance.

Future research will focus on integrating real-time demand response, predictive scheduling using AI models, and robust optimization under resource and price uncertainties to further enhance system resilience and scalability.

**Table 7:** Load sensitivity (Up to 125% – Case 1).

Load	ASC ($)	$$\Delta$$ASC (%)	Fuel Cost ($)	Emission Cost ($)	RF (%)	LPSP (%)
100%	588872.57	0.00	66850.04	2716.48	69.94	0.00
105%	595401.08	1.11	71810.26	3312.15	63.34	0.00
110%	600956.96	2.05	76770.48	3907.81	56.75	0.00
115%	606512.84	2.99	81730.70	4503.47	50.16	0.00
120%	612068.73	3.94	86690.92	5099.14	43.57	0.00
125%	617624.61	4.88	91651.14	5694.80	36.97	0.00

**Table 8:** DG load reduction (-25% in 5% Steps – Case 1).

% of DG Power	ASC [$]	$$\Delta$$ASC [%]	Fuel [$]	Emis. [$]	RF [%]	LPSP [%]
0%	588872.57	0.00	66850.04	2716.48	69.93	0.00
-5%	591112.06	0.38	67981.08	2852.31	69.93	0.00
-10%	592378.92	0.59	69112.12	2988.13	69.93	0.00
-15%	593645.79	0.81	70243.16	3123.96	69.93	0.00
-20%	594912.65	1.03	71374.20	3259.78	69.95	0.00
-25%	596179.52	1.24	72505.24	3395.61	69.98	0.00

**Table 9:** Case 1: wind speed decrease (-20% in 5% Steps).

Wind speed	ASC [$]	$$\Delta$$ASC [%]	Fuel [$]	Emis. [$]	RF [%]	LPSP [%]
0%	588872.57	0.00	66850.04	2716.48	69.94	0.00
-5%	598121.51	1.57	74239.03	3603.81	55.77	0.00
-10%	605973.39	2.90	81249.08	4445.64	39.15	0.00
-15%	613400.79	4.17	87880.17	5241.95	19.48	0.00
-20%	620403.69	5.35	94132.27	5992.75	7.56	0.00

**Table 10:** Case 1: wind speed sensitivity (increasing trend).

Wind speed	ASC [$]	$$\Delta$$ASC [%]	Fuel [$]	Emis. [$]	RF [%]	LPSP [%]
100%	588872.57	0.00	66850.04	2716.48	69.94	0.00
105%	583069.09	0.98	60800.42	1990.00	80.04	-2.00
110%	576542.55	2.93	54973.62	1290.27	88.21	-4.00
115%	572116.09	2.84	51021.73	815.69	93.18	-9.00
120%	569292.90	3.32	48501.22	513.02	96.06	-15.0

**Table 11:** Case 2: load sensitivity (up to 125% in 5% steps).

Load	ASC [$]	$$\Delta$$ASC [%]	Fuel [$]	Emis. [$]	RF [%]	LPSP [%]
100%	141367.30	0.00	66867.46	2718.58	70.37	0.00
105%	146923.18	3.90	71827.67	3314.24	63.87	0.00
110%	152479.06	7.86	76787.89	3909.90	57.38	0.00
115%	158034.94	11.79	81748.11	4505.57	50.90	0.00
120%	163590.83	15.72	86708.33	5101.23	44.40	0.00
125%	169146.71	19.65	91668.55	5696.89	37.91	0.00

**Table 12:** Case 2: sensitivity to DG load decrease.

% of DG Power	ASC [$]	$$\Delta$$ASC [%]	Fuel [$]	Emis. [$]	RF [%]	LPSP [%]
0%	141367.30	0.00	66867.46	2718.58	70.37	0.00
-5%	142635.14	0.89	67999.37	2854.51	70.37	0.00
-10%	143902.98	1.79	69131.28	2990.43	70.37	0.00
-15%	145170.82	2.69	70263.19	3126.36	70.37	0.00
-20%	146438.66	3.58	71395.10	3262.29	70.42	0.00
-25%	147706.50	4.48	72527.02	3398.23	70.46	0.00

**Table 13:** Case 2: sensitivity analysis on wind power (PV/WIND/DG/Battery) (decrease to 25% in steps of 5%).

Wind speed	ASC [$]	$$\Delta$$ASC [%]	Fuel [$]	Emis. [$]	RF [%]	LPSP [%]
0%	141367.30	0.00	66867.46	2718.58	70.37	0.00
-5%	142184.71	0.58	67597.23	2806.21	69.12	0.00
-10%	142960.20	1.12	68289.58	2889.36	67.91	0.00
-15%	143693.78	1.65	68944.51	2968.01	66.75	0.00
-20%	144385.42	2.13	69562.00	3042.16	65.63	0.00
-25%	145035.14	2.59	70142.06	3111.82	64.57	0.00

**Table 14:** Case 2: wind resource enhancement (+125%).

Wind speed	ASC [$]	$$\Delta$$ASC [%]	Fuel [$]	Emis. [$]	RF [%]	LPSP [%]
100%	141367.30	0.00	66867.46	2718.58	70.37	0.00
105%	140509.27	0.60	66101.42	2626.58	71.65	0.00
110%	139758.44	1.13	65431.09	2546.08	72.81	0.00
115%	138985.54	1.68	64741.06	2463.22	73.97	0.00
120%	138184.41	2.25	64025.81	2377.33	75.15	0.00
125%	137389.73	2.81	63316.34	2292.13	76.31	-1.00

**Table 15:** Case 2: impact of reduced solar input.

Irrad.	ASC [$]	$$\Delta$$ASC [%]	Fuel [$]	Emis. [$]	RF [%]	LPSP [%]
0%	141367.30	0.00	66867.46	2718.58	70.37	0.00
-5%	145449.78	2.89	70512.24	3156.27	63.88	0.00
-10%	149509.15	5.76	74136.39	3591.49	56.74	0.00
-15%	153545.40	8.61	77739.91	4024.23	48.87	0.00
-20%	157558.55	11.41	81322.79	4454.49	40.12	0.00
-25%	161548.58	14.28	84885.04	4882.27	30.37	0.00

**Table 16:** Annual cost breakdown under different configurations.

Annual Cost	WIND [$]	PV & WIND [$]	DG only [$]
ASC	588872.57	**141367.30**	156782.85
ACC	293025.85	**44460.96**	1398.90
AFC	66850.04	**66867.46**	143433.63
AEC	2716.49	**2718.58**	11913.26
AOM	7760.82	**1177.55**	37.05
ARC	219492.01	**26142.75**	0.00

## Limitations and scope for future work

Although the proposed APO-based hybrid microgrid planning framework demonstrates strong performance, several limitations define the scope of the present study. The analysis relies on deterministic solar, wind, and load profiles, which do not capture stochastic variations, forecasting errors, or extreme weather events, and fixed economic parameters such as fuel price, emission cost, and interest rate are assumed without modeling their long-term variability. Battery degradation and diesel generator aging are represented using simplified formulations, and the technology mix is limited to PV, wind turbines, diesel generators, and lithium-ion batteries, excluding alternative storage technologies such as hydrogen systems, flow batteries, supercapacitors, or hybrid AC/DC architectures. Moreover, APO exhibits higher computational overhead than GWO, ALO, and SFOA due to its dual-stage search process, and the framework focuses solely on offline planning without integrating real-time control, predictive scheduling, or demand-response strategies. Future research should therefore incorporate uncertainty-aware optimization, dynamic economic modeling, advanced degradation mechanisms, broader technology portfolios, computational enhancements to APO, and real-time energy management to further improve system resilience, flexibility, and scalability.

## Conclusion

This paper introduced a comprehensive APO-based framework for the optimal sizing and dispatch of standalone hybrid microgrids integrating PV, wind turbines, diesel generators, and battery storage. By incorporating economic cost, CO$_2$ emissions, battery degradation, and reliability within a unified objective function, the proposed method enables a balanced and sustainable microgrid design.

The key contributions of this work can be summarized as follows. First, the proposed framework is one of the most comprehensive APO-based models developed for hybrid microgrid planning, jointly optimizing system sizing, renewable dispatch, diesel generator operation, and battery degradation within a unified techno-economic objective. Second, the study provides a rigorous benchmark against established metaheuristics (GWO, ALO, and SFOA) using real annual meteorological and load data, demonstrating clear improvements in annual system cost, renewable fraction, and reliability. Third, the approach integrates both economic and environmental indicators–including ASC, CO$_2$ emissions, EER, and LPSP = 0–achieving simultaneous enhancement of cost-effectiveness and sustainability. Finally, the framework is validated through detailed seasonal analysis and extensive sensitivity studies, confirming its robustness under significant variations in load demand and renewable resources.

Simulation studies using real meteorological and demand data from Ras Ghareb, Egypt, demonstrate that APO consistently outperforms benchmark metaheuristics (GWO, ALO, and SFOA), achieving higher renewable penetration, lower annual system cost, and zero unmet energy demand across all scenarios.

In both case studies, APO exhibited fast and stable convergence–typically within 30 iterations–while effectively adapting system operation to seasonal variations. Winter operation showed over 85% contribution from wind energy, summer conditions highlighted controlled diesel reliance during peak periods, and spring results achieved more than 94% renewable supply. Sensitivity analyses further showed that the optimized configuration maintains stable performance under $\pm 20\%$ variations in load, wind resources, and solar irradiance. These findings confirm the robustness and reliability of the APO-based design for real-world microgrid environments.

Although APO incurs moderately higher computational time per run than GWO, ALO, and SFOA, its complexity remains comparable in order, and the additional cost is justified by the consistently superior economic and environmental performance in offline planning scenarios.

Beyond technical performance, the proposed APO-based planning framework also offers practical value for microgrid deployment in remote and underserved regions. The ability to achieve high renewable penetration, stable operation, and minimized diesel reliance aligns with Egypt’s and Africa’s broader policy goals for energy security and rural electrification. As such, the results can support policymakers and developers in designing cost-effective, low-emission microgrids suitable for real-world field implementation.

Looking ahead, several opportunities exist to further strengthen the framework. Incorporating stochastic solar and wind models, probabilistic load forecasting, or robust optimization could enhance resilience under uncertain conditions. Integrating predictive or adaptive controllers may improve real-time dispatch decisions, while modular design strategies could support scalability across larger or interconnected microgrids. Future work will therefore focus on coupling APO with data-driven forecasting, adaptive control schemes, and multi-microgrid coordination under uncertainty to expand its applicability and real-world impact.

## Data Availability

The datasets used and/or analyzed during the current study are available from the corresponding author upon reasonable request.
